# Lip-Reading Aids Word Recognition Most in Moderate Noise: A Bayesian Explanation Using High-Dimensional Feature Space

**DOI:** 10.1371/journal.pone.0004638

**Published:** 2009-03-04

**Authors:** Wei Ji Ma, Xiang Zhou, Lars A. Ross, John J. Foxe, Lucas C. Parra

**Affiliations:** 1 Department of Neuroscience, Baylor College of Medicine, Houston, Texas, United States of America; 2 Department of Biomedical Engineering, The City College of New York, New York, New York, United States of America; 3 Program in Cognitive Neuroscience, Department of Psychology, The City College of New York, New York, New York, United States of America; 4 The Cognitive Neuroscience Laboratory, Nathan S. Kline Institute for Psychiatric Research, Program in Cognitive Neuroscience and Schizophrenia, Orangeburg, New York, United States of America; 5 Program in Neuropsychology, Department of Psychology, Queens College of the City University of New York, Flushing, New York, United States of America; University of California Davis, United States of America

## Abstract

Watching a speaker's facial movements can dramatically enhance our ability to comprehend words, especially in noisy environments. From a general doctrine of combining information from different sensory modalities (the principle of inverse effectiveness), one would expect that the visual signals would be most effective at the highest levels of auditory noise. In contrast, we find, in accord with a recent paper, that visual information improves performance more at intermediate levels of auditory noise than at the highest levels, and we show that a novel visual stimulus containing only temporal information does the same. We present a Bayesian model of optimal cue integration that can explain these conflicts. In this model, words are regarded as points in a multidimensional space and word recognition is a probabilistic inference process. When the dimensionality of the feature space is low, the Bayesian model predicts inverse effectiveness; when the dimensionality is high, the enhancement is maximal at intermediate auditory noise levels. When the auditory and visual stimuli differ slightly in high noise, the model makes a counterintuitive prediction: as sound quality increases, the proportion of reported words corresponding to the visual stimulus should first increase and then decrease. We confirm this prediction in a behavioral experiment. We conclude that auditory-visual speech perception obeys the same notion of optimality previously observed only for simple multisensory stimuli.

## Introduction

Vision often plays a crucial role in understanding speech. Watching a speaker's facial movements, especially lip movements, provides input that can supplement the information from the speaker's voice. “Lip-reading” or “speech-reading” allows hearing-impaired individuals to understand speech (e.g. [1], [2]), and in subjects with intact hearing abilities, substantially facilitates speech perception under noisy environmental conditions [3], [4], [5], [6], [7]. This benefit has been quantified by measuring performance enhancement due to visual input as a function of auditory noise [8], [9], [10], [11]. In these experiments, participants were asked to identify spoken words from a checklist, delivered during an auditory-alone condition and during an auditory-visual condition in which the speaker's face was visible. The benefit from the visual information, measured in percent correct, was found to be greatest when the auditory stimulus was most noisy (but see [12], [13]). This seems to be evidence for inverse effectiveness, a widely cited concept stating that the largest multisensory enhancement is expected when a unisensory stimulus is weakest [14]. However, when multisensory word recognition was tested under more natural conditions (without a checklist), maximal gain was found not at low, but at intermediate signal-to-noise ratios (SNRs) [15], in apparent contradiction to inverse effectiveness.

Here, we first replicate and extend the findings by Ross et al. [15]. We then examine human performance when veridical visual speech information is replaced by purely temporal visual information and find that a minimum sound quality is required for such visual input to improve performance. This is again inconsistent with inverse effectiveness. We formulate a Bayesian cue integration model that explains these behavioral findings. In Bayesian cue integration, the relative reliabilities of cues are taken into account when inferring the identity of the source stimulus. For simple stimuli, human multisensory integration has been shown to be close to Bayes-optimal (e.g. [16], [17], [18]). For identification tasks using multidimensional stimuli such as speech, the Bayesian model predicts visual enhancements that are largest at intermediate auditory SNRs, provided that a sufficiently large vocabulary is used. Inverse effectiveness is predicted only when the underlying feature space is low-dimensional. To further test the Bayesian theory, we generate a prediction for the perceptual integration of slightly incongruent auditory and visual stimuli: at very low SNR, the percentage of reported words that match the visual stimulus should increase as SNR increases, even though the weight to vision decreases. We report behavioral data confirming this counterintuitive prediction. Together, these results suggest that Bayesian optimality of cue integration is not limited to simple stimuli.

## Methods

### Psychophysics

#### Subjects

Thirty-three volunteer subjects (14 female) were recruited among the student population at CCNY and gave informed consent (written) in accordance with the guidelines of the IRB at CCNY. Seventeen subjects participated in the first experiment, which only contained matching auditory and visual stimuli (congruent), while 16 subjects participated in the second experiment, which also included conflicting auditory-visual stimuli (incongruent). Subjects were native American-English speakers or learned English when they were young. All participants had normal or corrected-to-normal vision and reported normal hearing.

#### First experiment

**Figure 1:**
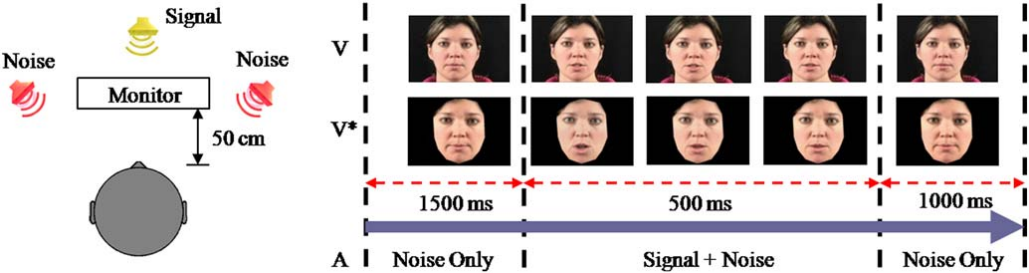
Experimental set-up and timing of audio-visual stimuli.

Auditory (A) and auditory-visual (AV) stimuli were the same as in [15]. 546 Simple monosyllabic English words were selected from a well-characterized normed set based on their written-word frequency [19]. These high-frequency words, uttered in isolation by a female American-English native speaker, were recorded as audio and video, and reproduced to subjects as audio alone (A) or as audio and video together (AV). Stationary acoustic noise with a 1/*f*-spectrum and a frequency range of 3 Hz to 16 kHz was presented, extending 1.5 s before and 1 s after the speech sound. Video was presented on a 17-inch LCD monitor with the face extending a visual angle of approximately 15°. Speech sound was played back from a centrally located loudspeaker, and the noise from two lateral speakers, all at 50 cm distance from the subject (see Figure 1). This configuration was originally chosen to allow for spatial auditory cues that may interact with visual cues. The A condition included a stationary visual stimulus to indicate to the subject the onset and offset of the speech sound (face with mouth closed or mouth open). This controlled for a bias in attention, which may otherwise favor the AV condition, since the video may give the subject a clue as to when to attend to the auditory stimulus (this contrasts the experiment in [15] which did not indicate speech onset in the A condition). Speech was presented at a constant 50 dB sound pressure level and noise at levels between 50 dB and 74 dB in steps of 4 dB, resulting in an SNR ranging from 0 dB to −24 dB. To generate the modified video sequence (V* stimulus, AV* condition) we used a video synthesis program that can generate a face which is similar in appearance to a given natural face [20]. We used natural faces instead of artificial visual stimuli as they are known to generate the largest auditory-visual enhancements in speech recognition [21]. The method used features extracted from the clean audio signal to generate articulations of the mouth, eyes, brows, and outline of the face. From these, realistic video frames were generated (for details, see the Supporting Information and Figure S1). Here we used the power of the audio (in time frames of 40 ms) as the only feature to generate the video. Hence, the V* stimulus can only represent visual information associated with the overall intensity fluctuations of the signal in time. The video cannot reflect any information associated with the detailed spectral content of the original speech signal. It may, at most, convey broad phonetic characterizations such as vowel versus consonant (vowels tend to have higher energy content).In each of the 3 conditions (A, AV, AV*), 26 words were presented at each of 7 SNR levels. Each word was presented only once, resulting in a total of 546 words (26×7×3). Stimuli were identical for all subjects to reduce cross-subject variability.

#### Second experiment

The second experiment, which included incongruent stimuli, included four conditions: visual-only (V), auditory-only (A), congruent auditory-visual (A = V), and incongruent auditory-visual (A≠V). Auditory and visual stimuli were selected from the same set of words as above. The A≠V condition presented the sound of one word while showing a face uttering a different but similar-sounding word. To select similar-sounding words, we computed the correlations of spectrograms of all word pairs within a set of 700 words and selected pairs with the highest correlation. As before, the 700 words were selected as the most frequent monosyllabic words, following [19]. Words with homophones were excluded. Words were presented only once, either as video or as audio. The V condition was presented with no sound, while the A condition was presented with a static visual stimulus as above, to control for attention. The noise had the same timing and spectral characteristics as above, but SNR was now adjusted in the range of −28 dB to −8 dB by varying the level of the speech signal and keeping the noise at a constant 50 dB (the intention was to help subjects maintain an equal level of effort despite the low SNR in some of the trials). Fourteen subjects were also tested with pure auditory noise (−∞ dB). No significant behavioral difference was found between −28 dB and this pure-noise condition, suggesting that at this lowest noise level, speech is fully masked by the noise. The −28 dB condition was included to capture the predicted increase of visual reports at low SNR. Higher SNR conditions were omitted to limit the total duration of the experiment. To prevent subjects from noticing that stimuli were incongruent, the A≠V condition was tested only up to −12 dB.

Each of the 4 conditions (A, V, A = V, A≠V) included 40 trials at each SNR level. In the A≠V condition, this made for a total of 400 words used (5 SNR levels×40 trials×2 words per trial). There were no repetitions among these 400 words. For the 6 SNR levels of the A and the A = V conditions, as well as the V-alone condition, a total of 520 words were drawn from the same pool (6×2+1 = 13 combinations of condition and SNR level; 40 trials each; 13×40 = 520). There were no repetitions among these 520 words, but overlap with the 400 words in the A≠V condition could not be avoided. Stimuli were identical for all subjects to reduce cross-subject variability.

#### Procedures

Except for varying SNR levels, the noise was identical in all conditions in order to reduce variability, while the presentation of all stimuli was fully randomized to control for potential learning effects. A brief instruction was shown to participants before the experiment. Participants were required to write down the words they identified, and asked to note when they did not recognize a word. Subjects had no time constraints to give their response, but answered in 5–10 seconds, making each experiment last approximately 90 minutes. For classification as correct, we insisted on correct spelling. After the experiment, participants were presented with the full list of words used in the experiment and asked to indicate any words they did not know. These words were then excluded from the analysis and no longer presented to subsequent subjects.

### Model

#### Background: words as points in a high-dimensional feature space

Traditionally, words are conceived of as a sequence of phonemes, with phonemes representing the elementary carriers of word identity. Classic phonetic features are grouped in categories such as place of articulation, voicing, and manner. These phonetic features have been derived empirically focusing on auditory stimuli. However, the definition of relevant phonemes depends also on the type of stimulus [22], [23]. For instance in speech-reading, the visual stimulus may not be sufficient to disambiguate among distinct phonemes (e.g. in words such as ‘pad, ‘bat’, and ‘mat’, the phonemes /p/, /b/, and /m/ are difficult to disambiguate visually and may be considered the same ‘viseme’ [24]). Similarly, an auditory stimulus distorted by noise, or degraded due to hearing loss will no longer communicate some phonetic features [25], [26]. The specific phonetic identification depends therefore on the specifics of the audio-visual speech stimulus. Given this dependence on the stimulus, there has been an effort to automatically extract relevant auditory and visual features directly from the stimulus in conjunction with behavioral experiments on phoneme identification [22], [27]. These experiments, and the associated computational and modeling approaches, by and large have converged on the notion that words can be represented by a conjunction of features, with each phoneme in a word contributing a set of features. This feature space can be generally thought of as a topographic space with well-defined neighborhood relationships [28]. For instance, words that are “close by” are more likely to be confused when the stimulus is distorted by noise. In this feature space, words are not evenly distributed, and words that are clustered in high-density regions are harder to recognize [28], [29]. The conjunction of phonetic features of several phonemes can make this space rather high-dimensional. However, not all phonetic combinations occur equally likely, and even fewer combinations represent actual words in a lexicon [30]. Such phonotactic and lexical constraints allow accurate word identification even in a reduced phonetic representation [23], [29], [31]. Essentially, in the high-dimensional joint feature space, many areas have a zero probability of containing a lexically correct word. Empirical evidence also suggests that high-frequency words are easier to recognize, implying that the prior probability of a given word plays a role in correct identification [28].

#### Bayes-optimal word recognition in n-dimensional space

We present a first-principles model for multisensory word recognition that captures the main concepts of a stimulus neighborhood in high-dimensional feature space, where the reliability of the signal affects the size of the neighborhood and lexical information is represented by the distribution of words.

Let us assume that there are *n* features and that the observer's vocabulary can be represented by points in this *n*-dimensional space, which we will call word prototypes. Different speakers, different articulation, and noise will induce variability in the perceived stimulus for a given word. We assume that these noisy examplars of the word are represented in the observer's brain within some neighborhood of the prototype. We characterize their distribution by a *n*-dimensional normal distribution centered at the prototype. An important distinction from previous models is that we do not differentiate explicitly between visual and auditory features. Both the auditory and visual stimulus contribute to the observer's estimate for each feature dimension. The variance associated with these estimates may differ for the auditory versus the visual stimulus. In this view, a feature that is primarily auditory is characterized by a smaller variance afforded by the auditory than by the visual stimulus. Moreover, we will allow for correlated features.

The process of word identification is modeled as follows (for details, see the Supporting Information). First, we define the generative model, also called noise model. For a given vocabulary size *N*, word prototypes are denoted by vectors 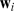 (*i* = 1‥*N*) and are randomly drawn from a *n*-dimensional normal distribution. On each trial, a test word 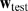 is presented to the subject and gives rise to noise-perturbed exemplars 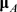 and 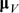 in the subject's brain. These are sampled from Gaussian distributions with mean at 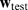 and covariance matrices 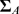 and 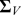, respectively (which do not depend on 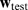). We model the overall level of reliability of the stimuli by non-negative scalars, *r_A_* for auditory and *r_V_* for visual. These parameters are usually under experimental control – for example, increasing the auditory signal-to-noise ratio leads to an increase in *r_A_*. The covariance matrices 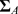 and 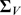 are scaled by factors of 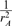 and 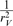, respectively. The equivalent of such a scaling in one dimension would be that reliability is inversely proportional to the standard deviation of the noise distribution, and therefore closely related to the 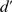 measure.

**Figure 2:**
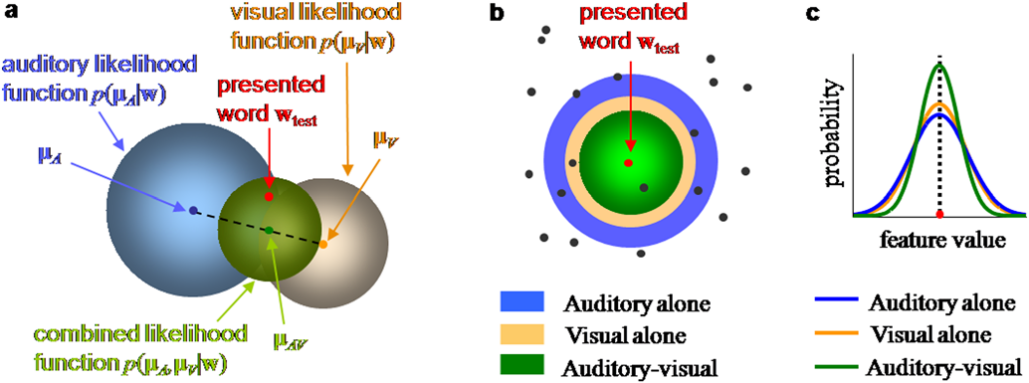
Bayesian model of auditory-visual word recognition. a. Inference process on a single multisensory trial. Word prototypes are points in a high-dimensional space (of which two dimensions are shown). The presented word (in red) gives rise to an auditory (μ*_A_*) and a visual (μ*_V_*) observation (which are the respective unisensory estimates if only one modality is presented). Based on these, the brain constructs likelihood functions over utterances w, indicated by muted-colored discs. The diameter of a disc is proportional to the standard deviation of the Gaussian. The auditory-visual likelihood is the product of the unisensory likelihoods and is centered at μ_AV_ (see text), which is the multisensory estimate on this trial. b. Across many repetitions of the test word, the estimates will form a distribution centered at the test word. The estimate distributions are shown as bright-colored discs for the auditory-alone (A), visual-alone (V), and auditory-visual (AV) conditions. Since the distributions “cover” many words, errors will be made. Note the different interpretations of the discs in a and b: single-trial likelihood functions, versus estimate distributions across many trials. c. Side view of the estimate distributions in b. The AV estimate distribution is sharper than both the A and the V distribution, leading to fewer errors. This indicates the advantage conferred by multisensory integration.

Having specified the generative model, we can now formalize the Bayesian inference process which “inverts” it. To the subject's nervous system, the exemplars 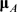 and 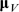 are known, while 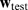 is not; for the experimenter, the reverse holds. On each multisensory trial, 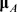 and 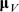 provide the brain with a likelihood function 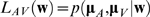, i.e. a function over (not necessarily lexically correct) utterances **w**, indicating how probable it was that each has given rise to 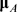 and 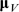. Assuming that auditory and visual noise are independent, this likelihood function is the product of both unisensory likelihood functions, i.e. 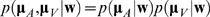 (see Figure 2a). The unisensory likelihood functions are defined by the noise model outlined above, and consequently, the multisensory likelihood function will also be an *n*-dimensional Gaussian, with mean at 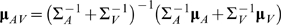 and covariance matrix 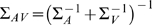. The utterance 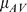 is the multisensory maximum-likelihood estimate. The well-known one-dimensional analogs of these expressions are 
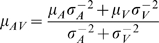
 and 
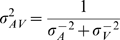
 (e.g. [17]). The former means that the multisensory likelihood function will have its maximum closest to the peak of the likelihood function corresponding to the most reliable modality; the linear weights of both modalities are determined by their reliabilities. (Interestingly, **μ** *_AV_* does not necessarily lie on the line connecting 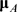 and 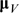.) The latter indicates that the multisensory likelihood function is narrower than both unisensory likelihood functions, indicating the benefit of combining both modalities.

Still on a single trial, we account for uneven word frequencies by multiplying the likelihood values of all utterances with prior probabilities. These are taken to be zero for non-lexical utterances and are assigned according to an exponential distribution for lexical words, 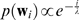, with a decay constant 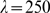. Previous studies do not provide strong guidance on how to choose this prior. It is most likely a combination of frequency knowledge acquired before and during the experiment. Good fits to the data are possible with a variety of priors we have tried. This issue deserves further attention. Posterior probabilities are computed for all words in the vocabulary through 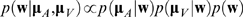. According to the model, the observer then reports the word with maximum posterior probability (for details, see Supporting Information). Trials for which the reported word was equal to the test word were counted as correct. The “correctness regions” for each word typically have heterogeneous and irregular boundaries.

In the generation of the word prototypes as well as the generation of noisy word exemplars we sampled from normal distributions. The *k*-dimensional correlation structure in the corresponding covariance matrices was generated by adding to the diagonal matrix a product, 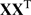, of a *n×k*-dimensional matrix **X** with normally distributed coefficients and an adjustable scale.

Across many trials, the maximum-likelihood estimates (either auditory, visual, or auditory-visual) of a given word form a probability distribution, as illustrated in Figure 2b. It turns out that when all distributions are Gaussian, the covariance matrix of this distribution is equal to that of a corresponding single-trial likelihood function (A, V, or AV). Therefore, estimation precision is governed by stimulus reliability, and many papers only discuss the estimate distributions. However, it is important to keep in mind that a full likelihood function is encoded on a single trial. This is particularly important when the prior distribution is not uniform.

#### Fitting the models to the behavioral data

To relate the Bayesian model to the behavioral data we have to identify the relationship between auditory reliability 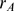 and SNR. As SNR increases, the reliability of the auditory signal increases monotonically. Here, we simply assume a rectified linear relationship between SNR measured in dB (a logarithmic scale) and reliability: 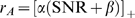, where α and β are constants and [·]_+_ sets negative arguments to zero. The data is fit by first optimizing α and β in the A condition. The AV and AV* conditions are then fit by adjusting visual reliability 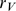 separately for each. Throughout this paper, we plot performance as a function of SNR when behavioral data are fitted, and as a function of 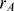 otherwise (as this is more general).

The percentage of correct identification was computed by testing over a large number of test words. Behavioral performance was fit to the model performance by using 1000 test words per data point (not to be confused with the number of vocabulary words *N*). The final performance curves according to the model were computed with 8000 test words per data point, to produce smoother traces.

## Results

### Summary of results

**Figure 3:**
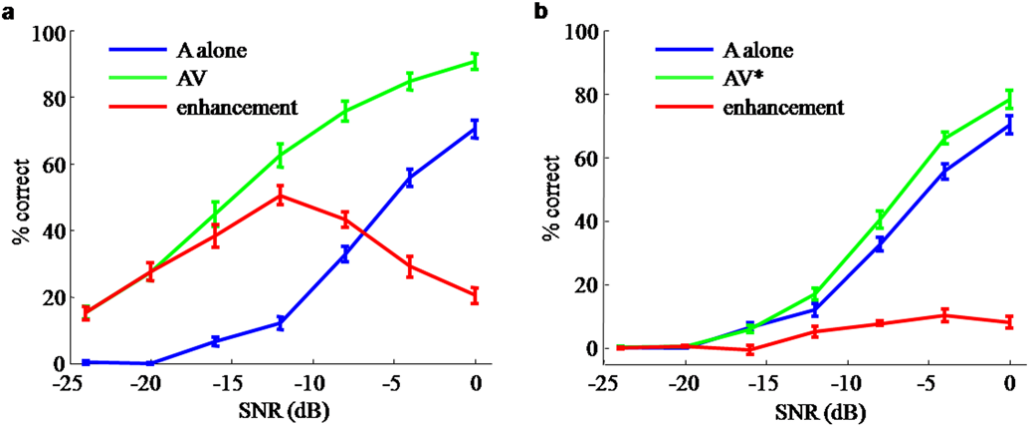
Behavioral performance in open-set word recognition. Data consisted of auditory-alone performance (blue) and auditory-visual performance (green). The multisensory enhancement (red) is the difference between auditory-visual and auditory-alone performance. Error bars indicate s.e.m. a: Full visual information (AV). b: Impoverished visual information (AV*). In both cases, maximum enhancement occurs at intermediate values of auditory SNR.

We first present the results of our behavioral experiment, showing that open-set word identification in noise does not follow inverse effectiveness (Figure 3). In both the AV and the AV* condition, the enhancement due to additional visual information is maximal not at the highest but at an intermediate or low noise level.

**Figure 4:**
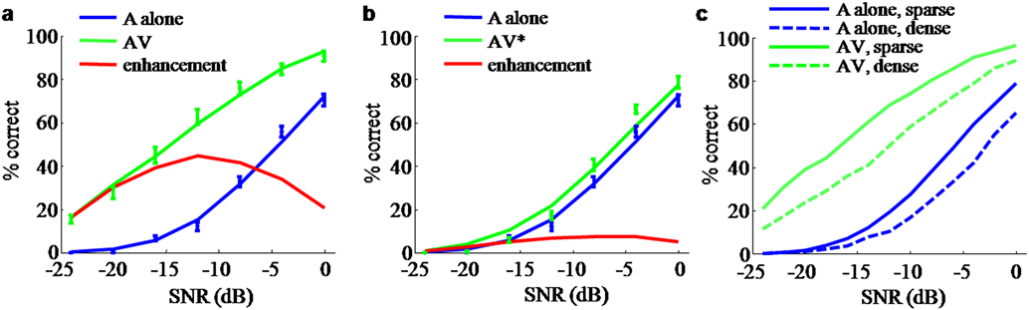
A Bayesian model of speech perception can describe human identification performance. A vocabulary of size *N* = 2000 was used. Words were distributed in an irregular manner in a space of dimension *n* = 40. For details of the fits, see the Supplemental Material. a: Data (symbols) and model fits (lines) for A-alone and AV conditions. The red line is the multisensory enhancement obtained from the model. b: Same for impoverished visual information (AV*). c: Words in high-density regions are harder to recognize. In the simulation in a, words were categorized according to their mean distance to other words. When the mean distance is large (sparse, solid lines), recognition performance in both A-alone and AV conditions is higher than when the mean distance is small (dense, dashed lines).

**Figure 5:**
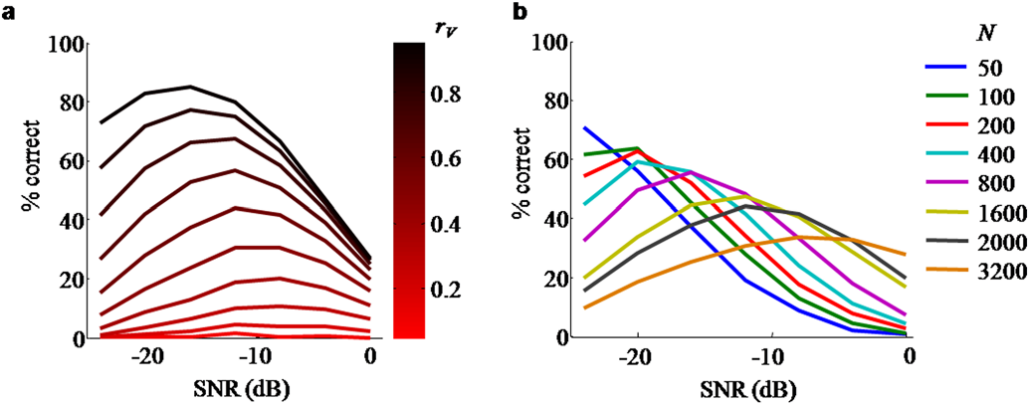
Predictions of the Bayesian model for auditory-visual enhancement as a function of auditory SNR, for various values of: a: visual reliability (from 0.05 to 0.95 in steps of 0.10); b: vocabulary size. For both plots, all other parameters were taken from the fit in Figure 4. See Results for interpretation.

We then present results of the model that conceives of speech recognition as a Bayesian cue combination process. This model implements the key statistical properties of phonetic features and lexical information that are known to affects human speech recognition performance. The computations show that Bayesian inference produces multisensory enhancements that do not decline monotonically with SNR but have a maximum at intermediate SNR. The resulting performance curves are shown to fit the present behavioral data with high accuracy (Figure 4a). We next modeled the auditory-visual enhancement when visual reliability is reduced and find that the model effects are consistent with the behavioral results of the impoverished visual condition AV* (Figures 4b and 5a). We show that words in higher-density regions are harder to recognize (Figure 4c), consistent with earlier findings. We also show that when vocabulary size is reduced, the enhancements resemble earlier behavioral data on speech perception in noise, which used checklists instead of an open word set (Figure 5b).

**Figure 6:**
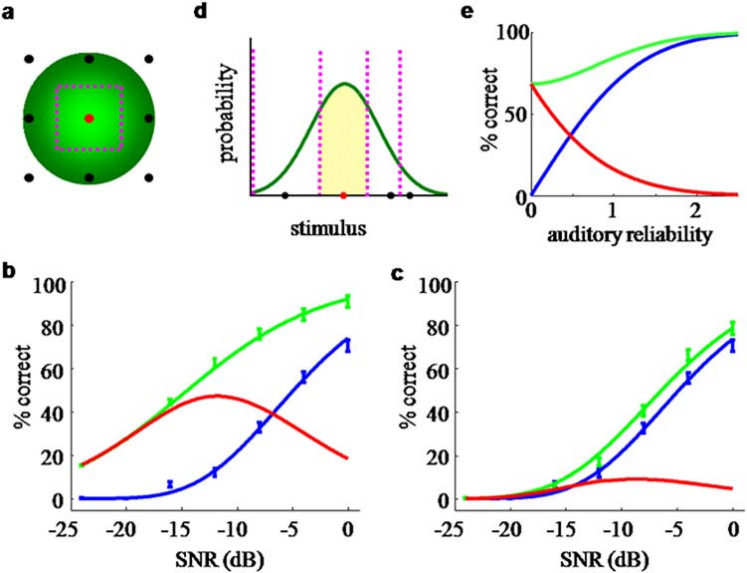
Optimal cue combination in multiple dimensions according to a simple analytical model. a. In this simplified model, word prototypes (dots) lie on a rectangular grid, here shown in two dimensions. The green blob indicates an example estimate distribution (compare Fig. 2b). The dashed lines enclose the correctness region when the central word is presented. b and c. The model was fitted to the data in the AV condition (b) and the AV* condition (c). Data are shown as symbols, lines are model fits. Colors are as in Fig. 3. d. The same model in 1 dimension, but now allowing word prototypes to be unequally spaced. The green curve is an estimate distribution. The vertical dashed lines are the boundaries of the decision regions. The shaded area corresponds to correct responses when the presented stimulus is the one marked in red. e. Typical identification performance in 1 dimension, for the A (blue) and AV (green) conditions. The multisensory enhancement (red) decreases monotonically with auditory reliability. This is an instance of inverse effectiveness. For details, see the Supporting Information.

We then provide evidence that these numerical results are a robust property of the model and do not depend on specific parameter choices. In particular, we show that the predicted performance curves show the same trends when we compute rigorous analytic expressions for a strongly simplified high-dimensional model (Figure 6). Moreover, we show rigorously that in 1 and 2 dimensions, optimal cue integration does follow an inverse-effectiveness rule. This suggests that high dimensionality of the feature space is both necessary and sufficient for the results to hold.

**Figure 7:**
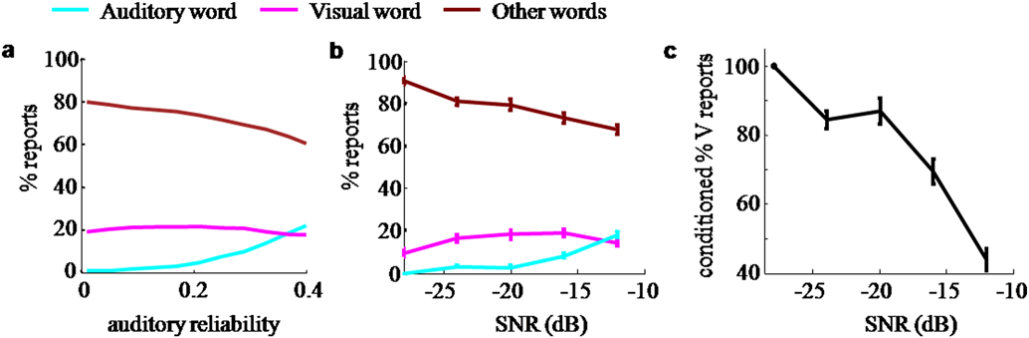
Effect of an auditory word on reports of an incongruent visual word. a. Illustration of the Bayesian prediction. An experiment was simulated in which pairs of slightly incongruent auditory and visual words are presented. On each trial, the observer integrates the signals and reports a single word. Frequencies of reporting the auditory word (cyan), the visual word (magenta), and other words (brown) are shown as a function of auditory reliability. As auditory reliability increases, the percentage reports of the visual word reaches a maximum before it eventually decreases. This is a small but significant effect. Note that the interpretation of both curves is completely different from that of Figures 3–4 (here, the only condition is multisensory, and there is no notion of correctness). A vocabulary of size *N* = 2000 and dimension *n* = 30 were used, and visual reliability was fixed at *r_V_* = 0.5. Robustness of the effect across dimensions and vocabulary sizes is demonstrated in Figure S5. b. Experimental test of the Bayesian prediction. The percentage reports of the visual word exhibits a maximum as a function of SNR. The curves in a have not been fitted to those in b. c. Reports of the visual word as a percentage of the total reports of either the auditory or the visual word, computed from the data shown in b. As expected, this declines monotonically with SNR.

The modeling efforts conclude with predictions for the case of cue conflict (incongruence), i.e. visual and auditory stimuli that do not represent the same word (Figure 7a). Finally, we present results of a subsequent behavioral experiment which confirm these theoretical predictions (Figure 7b), lending further support to the hypothesis that human speech identification follows Bayes-optimal cue combination. The congruent trials in this experiment show unambiguously that multisensory integration occurs at all SNR levels.

### Behavioral performance in an open-set word identification task does not follow inverse effectiveness

Monosyllabic words were presented in an auditory (A) and auditory-visual (AV) condition under varying noise levels. Subjects responded in writing which word they identified. In addition to the original video, a modified video sequence was generated for each word and presented together with the corresponding original audio (AV*). The goal of this modified video sequence was to represent only temporal information, but not spectral information. The rates of correct identification are shown in Figure 3 and confirm previous literature on the benefits of auditory-visual speech in noise.

In the AV condition (Figure 3a), identification performance at all noise levels improves by adding the visual information, with the highest gains occurring at intermediate noise levels. The enhancements are large and statistically significant by any measure. When contrasting these results with the study by Ross et al. as well as our second experiment below, one can see that the specifics of the performance gains depend on the experimental protocol (see Figure S2). However, in all instances, the maximum gain for the AV condition is obtained at a SNR of approximately −12 dB.

The enhancements in the AV* condition (Figure 3b) are smaller and were tested for significance as follows. A repeated-measures 2-way ANOVA shows a significant effect of the stimulus condition (AV* vs A) with *F*(1,32) = 80.3 and a significant effect of SNR with *F*(6,224) = 524. This means that adding the V* visual stimulus improves performance significantly with respect to the A-only condition. It also means, trivially, that performance varies with SNR. The ANOVA analysis shows a significant interaction between the two factors (*F*(6,224) = 10.9), indicating that enhancement across the two conditions changes with SNR. (Compare this to the effect sizes in the AV vs A conditions, where we find *F*(1,224) = 510 for the difference between conditions, *F*(6,224) = 495 for the effect of SNR, and *F*(6,224) = 25.0 for the interaction between the two factors.) A subsequent sequential paired t-test on each SNR (with Holms' correction for multiple comparisons) shows that there is an enhancement at high (less negative) SNR (for −8 dB or higher) and no significant enhancement below −12 dB. Put differently, for the AV* condition, a minimum auditory SNR is required before the additional visual stimulus can aid word identification. This indicates that performance enhancements follow the opposite trend from what one would expect for inverse effectiveness. Significance in all these tests falls at a *p*-value of 0.001 or less, except for the gain due to V* at −12 dB, for which *p*<0.01.

### Bayes-optimal cue combination in high dimensions predicts largest multisensory enhancement at intermediate noise levels

Speech recognition is a process in which perceived phonetic information is compared to a mental lexicon. Here we use a model that is broadly consistent with results from linguistics which describe how phonetic features are be integrated with lexical information (see Methods). Briefly, the model regards word recognition as a Bayesian inference process in which vocabulary words are prototypes defined by a conjunction of phonetic features. A specific word stimulus corresponds to a point in this space and different instantiations of the same word are distributed in some proximity of the mean prototype (see Figure 2a). To mimic the varying similarity or distinctiveness of vocabulary words the prototypes were chosen to be unevenly distributed in this feature space, with close-by prototypes representing similar words. Each prototype word is assigned a prior likelihood to be observed thus capturing the uneven frequency of occurrence of different words in natural speech. In addition, we allow features to be correlated, which relaxes restrictions of previous models that often implicitly assume phonemes to be independent [28], [32].

We computed identification performance simulating auditory-only and auditory-visual stimulation for various values of auditory and visual reliability. The results of the model are shown in Figure 4 with suitably chosen parameters. To compare the results to behavioral data, they are plotted here as a function of auditory SNR. A rectified-linear relation between auditory reliability and SNR was assumed, with parameters determined by fitting the performance curve to the auditory-alone conditions. As SNR is increased, the model shows that performance reaches a maximum and ultimately decreases. Indeed, the model replicates the behavioral data with high accuracy (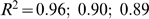 for conditions A, AV, and AV* respectively). For this example, we chose *N* = 2000 words as an estimate of the number of uniquely spoken monosyllabic words that may be known by our subject population (John Lawler, personal communication), 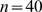 dimensions, and uncorrelated features. This left only 4 free parameters: 2 for the relation between auditory SNR and reliability, and 2 for the visual reliabilities in the AV and AV* conditions. Dimensionalities between 20 and 50 and vocabulary sizes of 800–3000 words give equally good results (see Supporting Figure S3a). This makes it impossible to reliably determine the parameter values from these data, but it speaks in favor of the generality of the qualitative conclusion that the behavioral data can be explained by a Bayesian model as long as dimension and vocabulary size are sufficiently high. Also note that a small vocabulary size or a low feature space dimension cannot account for the data. Finally, we tested several cases of nonzero correlations of various ranks between features in the auditory or visual noise; these correlations had little or no effect on the reported performance curves.

### Words in higher-density regions are harder to recognize

In earlier work using related models, it was found that words with more neighbors are harder to recognize [28], [29]. In order to confirm that this is the case in the Bayesian model, we divided the vocabulary into two subsets according to the density of their neighbors. In the simulation used to fit the behavioral data (Figures 4a and 4b), each word has a roughly normal distribution of distances to other words. However, the mean of this distribution varies across words, with some words being in high-density and others in low-density regions. We defined the subsets by whether the mean distance of a word to other words is larger or smaller than the median mean distance. We computed performance separately for each subset and found that indeed, for both A and AV conditions, performance is better on words with a higher mean distance to other words (see Figure 4c).

### Largest multisensory enhancement shifts to higher SNR as visual reliability decreases

The simulations for the AV and AV* conditions shown in Figures 4a and 4b are identical except for the values of visual reliability, with 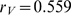 and 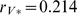, respectively. These values are consistent with the fact that the V* stimulus provides less reliable information. The auditory reliability at which maximum performance gain is attained depends on the reliability of the secondary modality. Figure 5a explores this behavior as a function of visual reliability. It shows that the maximum gain shifts to higher SNR as the reliability of the secondary modality increases. When the secondary modality is extremely uninformative, as in the AV* condition, the enhancement is very low at all SNR values and exhibits a maximum at high SNR. Therefore, we predict that subjects with an impaired ability to extract visual information will show their greatest multisensory enhancement at higher SNR than normal-vision controls.

### Largest multisensory enhancement shifts to higher SNR as vocabulary size increases

The Bayesian model explains why the maximum multisensory enhancement occurs at intermediate values of SNR. This raises the question what was different in earlier behavioral experiments that found the largest enhancement at the lowest values of SNR [8], [9], [33]. It was hypothesized before [15] that the number of words plays a crucial role, since in earlier studies the possible responses were restricted to a relatively short checklist. Therefore we checked in the numerical model the effect of vocabulary size on the multisensory enhancement function (see Figure 5b). Note that the vocabulary size is not the number of test words (which is kept constant), but the number of all monosyllabic words that the subject may consider in determining her response. All parameters were fixed at the values used in obtaining the fits of Figures 4a and 4b, except for the number of words in the vocabulary. We find that multisensory enhancement peaks at lower SNR as fewer words are considered. Therefore, with the vocabulary sizes used in earlier studies (e.g. at most 256 words in [8]), it is not surprising that inverse effectiveness was observed. When the maximum occurs at low SNR, but even lower levels of SNR are not used in the experiment, enhancement can appear to obey inverse effectiveness while in fact this is only a consequence of the limited SNR range used. The dependence of auditory-alone performance on set size is interesting in its own right [34] and warrants further attention in the context of the Bayesian model.

### Maximum enhancement at intermediate SNR is a generic property of Bayes-optimal cue combination in higher dimensions

The numerical modeling results replicate the behavioral data accurately, but does this depend critically on the specific modeling choices or the number of model parameters? Surprisingly, if we drop all the flexibility of the numerical model, and instead assume – unrealistically – that vocabulary words are uniformly distributed on a regular lattice of *n* independent features (see Figure 6a) we find that the main conclusions of the numerical model are preserved. This simplified case can be treated analytically. In Supporting Figures S4a–d, we show examples of the multisensory enhancement computed analytically, for different values of the dimension *n* and the visual reliability 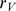. The curves replicate the observation that for higher dimensions the maximum performance gain is at intermediate values of auditory reliability (a mathematical proof is in the Supporting Information). It also confirms the numerical model result that higher auditory reliabilities are required to obtain maximal performance gain if the visual reliability is lower. Indeed, this simplified analytic model can explain the behavioral data with equally high accuracy (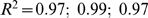 for A, AV, and AV* respectively; Figures 6b–c). Moreover, in the analytical model, it can be proven (see Supporting Information) that in 1 and 2 dimensions, maximum performance gain occurs at the lowest value of SNR, consistent with inverse effectiveness (see Figures 6d–e). In 1 dimension, the proof of this statement does not even require equal spacing of possible choices.

### Predictions for behavioral performance in multisensory cue combination

The value of the present model does not lie merely in explaining existing data, but also in its generality, which permits to make predictions about yet unobserved behavior. In the previous sections, we already discussed predictions regarding the location of the largest multisensory enhancement upon changing the visual reliability or the vocabulary size. These conclusions are consistent with known data but have not yet been fully experimentally tested.

Assuming Bayes-optimal behavior, we also predict that human performance in multisensory classification tasks will violate inverse effectiveness whenever the space of task-relevant features is high-dimensional. This is not limited to speech. For example, if an observer has to identify complex objects among an unconstrained number of alternatives based on noisy visual and tactile cues, the enhancement induced by the tactile cue should show a peak at intermediate values of image noise.

Finally, it would be worthwhile to test our prediction of inverse effectiveness for low-dimensional stimuli. Several behavioral studies in cats [35] and humans ([36], [37], [38], [39], [40], [41]; but see [42]) have claimed inverse effectiveness, but on different measures and in different conditions than the ones considered here.

### Prediction for incongruent auditory-visual cues

So far, we have considered the case where visual speech is congruent with auditory speech. Extensive literature exists on human behavior in the presence of an incongruence, or cue conflict, between auditory and visual speech. Massaro studied such conflict stimuli in the context of the McGurk effect [43] and found that it was well described by a Bayesian-type rule [5]. Many of these experiments were conducted using a factorial design based on nearby phoneme pairs such as /ba/-/da/. The present study raises the question how human performance can be described when the presented words are part of a much larger vocabulary, as in the experiment discussed here. For nearby word pairs, such as “dear”-“tear” or “pay”-“bay”, which in noise may be easily confused, subjects may not realize the incongruence of the auditory and visual stimuli. Hence, they will tend to merge the cues and when that happens, we expect the model of Bayesian cue integration to predict human behavior, without any need for further assumptions. Since there are now two sources (an auditory word and a visual word), there is no longer a notion of correctness, but trials will fall into three groups: those on which the auditory word is reported, those on which the visual word is reported, and those on which a different word (distracter) is reported.

**Figure 8:**
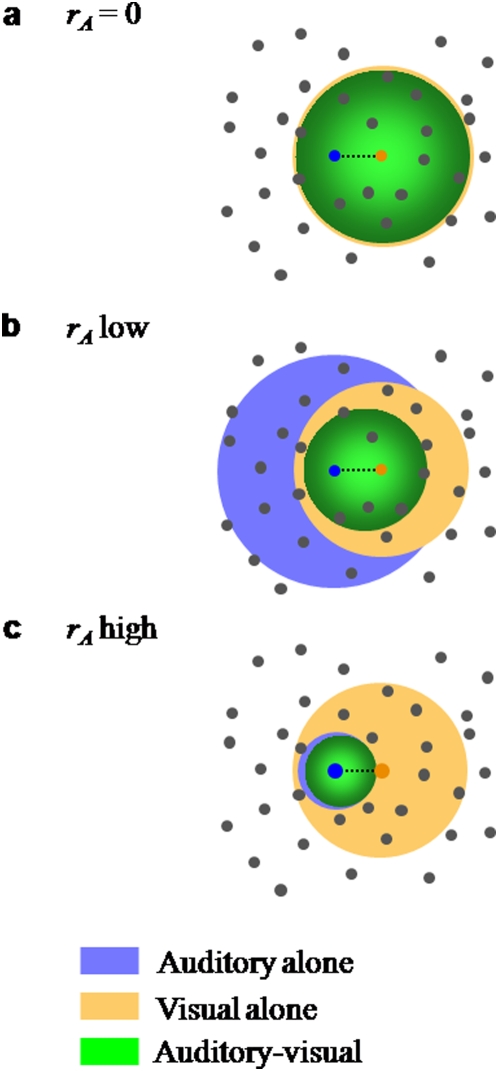
A large distracter set gets squashed. This figure illustrates the Bayesian model for integrating slightly incongruent auditory-visual stimuli. Dots represent word prototypes. The blue and orange dots represent the auditory and visually presented words, respectively. Each disc represents a Gaussian maximum-likelihood estimate distribution (A, V, or AV); its radius is proportional to the standard deviation of the Gaussian. a–c differ in auditory reliability but not in visual reliability. In a, auditory reliability is zero, therefore the V and AV distributions are identical. As auditory reliability increases, the AV distribution sharpens (thereby excluding more and more distractors) and shifts more towards the auditory word. These two effects together initially benefit both the auditory and the visual word, since the visual word is close to the auditory word and enjoys some of the increased probability mass (compare a and b). Eventually, the benefit will go more exclusively to the auditory word (compare b and c). This explains why in Figure 7b the percentage of reports of the visual word in the AV condition first increases and then ultimately decreases. Note that the auditory and the visual word do not have to be nearest neighbors.

The Bayesian model predicts (as many other models would) that when one keeps the visual noise level constant and increases auditory SNR, the frequency of reports of the auditory word will increase and the frequency of reports of other words will decrease. However, surprisingly, it also predicts that the frequency of reports of the visual word will first increase and then decrease, despite the fact that the weight to vision decreases throughout. This follows from a numerical simulation similar to those for the congruent case, and is illustrated for specific parameter choices in Figure 7a. This prediction holds across a wide range of vocabulary sizes and dimensions (see Figure S5) and is confirmed by the analytical model (see Supporting Information). It is a counterintuitive prediction, as one might expect the reports of the visual word to decrease monotonically as the weight to vision decreases. The reason that this does not happen is because as auditory reliability increases, two effects occur (Figure 8): 1) the mean of the distribution of auditory-visual maximum-likelihood estimates shifts towards the auditory word (this is what is meant by a decreasing weight to vision); 2) the estimate distribution narrows, leading to the squashing of a large distracter set. The interaction of both effects determines the frequency of visual reports. At very low SNR, the width of the distribution is large compared to the distance between the auditory and the visual word. Therefore, the stronger effect is the second one: the probability mass accumulates in the neighborhood of both presented words, which benefits both, since they are very close to each other. Only when the distribution becomes narrow compared to the distance between the two words, the enhancement will benefit the auditory word more exclusively. All this assumes that visual reliability is relatively poor, so that there is a strong tendency to integrate, even at the highest auditory SNRs used.

### Prediction on incongruent auditory-visual speech is confirmed by behavioral experiment

We tested the prediction for incongruent stimuli directly using the same set of auditory and visual words as in the first behavioral experiment. We selected words pairs based on the similarity of their spectrograms (see Methods). This resulted in pairs such as “cry-dry”, “smack-snake” and “lost-rust”. For the incongruent stimuli, one of the two words is presented as audio and the other as video. The prediction requires that subjects do not detect this mismatch and instead fuse the auditory-visual information into a common percept. To ensure this, we interleaved unisensory and congruent multisensory trials and limited the SNR on incongruent trials to at most −12 dB. Participants were informed of the incongruent condition only after the experiment. None of the subjects reported noticing an explicit mismatch between video and audio. The percentage of reported words that match the visual or auditory stimulus in the incongruent case (A≠V) are shown in Figure 7b. Evidently, the auditory reports increase with SNR, as expected. The trend for the visual reports seems to follow the prediction in Figure 7a. A one-way ANOVA comparing the percentages of visual reports shows that the difference across SNR is significant (*p*<0.02). Subsequent pairwise comparisons of the different SNR conditions confirm that visual reports at −28 dB and −12 dB are significantly lower than any of the intermediate SNR values (*p*<0.01 with Bonferroni correction). A simple quadratic fit to the data places the maximum at −19±7 dB (*R* ^2^ = 0.2 when including data for individual subjects, *p*<0.005). As sound quality improves further, subjects are more likely to report correctly what they heard and thus the number of visual reports decreases. This obvious expectation is indeed confirmed here at an SNR above −19 dB. The surprising prediction of the model, however, is that at the lowest SNR levels the trend should be reversed: the number of correctly reported visual words increases with increasing auditory reliability. This is indeed confirmed by the behavioral performance for SNRs below −19 dB.

To verify that the increasing frequency of visual word reports is due to the suppression of distracters and not to increasing weight to vision, we plotted the frequency of visual word reports conditioned on the observers reporting either the auditory or the visual word (see Figure 7c). This ignores all distracters and only considers visual relative to auditory word reports. As expected, this shows a monotonic decline with auditory SNR.

### Audio-visual integration occurred at all SNR levels

In the AV condition of the first experiment, identification performance at all noise levels improves by adding visual information. But at the same time, the AV performance is significantly greater than pure lip-reading performance at all SNR levels (*p*<0.01, corrected for multiple comparisons) if we assume the 7% measured for the visual-only condition on this data by Ross et al. [15]. To confirm this result, the second experiment measured the visual-only condition explicitly, resulting in a recognition performance of 5.3±1.5% (see Figure S2b). A post-hoc paired *t*-test shows significant improvement over the V condition for the AV condition down to −28 dB (*p*<0.0001). Hence, in these experiments, even marginal auditory information seems to aid in lip-reading (compare [44], where voice pitch was used as an auditory cue) and multisensory integration is occurring at all SNR levels.

## Discussion

### A case for Bayesian optimality

The benefits of speech-reading are well-documented (for a review see [1]) and have been described with computational models [29], [32], [45]. The notion that words form a neighborhood relationship in some high-dimensional features space was captured also by Luce's Neighborhood Activation Model (NAM) [28], [29]. The model uses performance measures on individual phonemes to estimate the performance of identifying full words. Similar to the present work, it incorporates word frequency (prior likelihood) and expresses lexical information as permissible points in the joint feature space.

However, the present study is the first that puts the observed gains in the context of optimal inference. This work was based on the recent finding that maximum auditory-visual gain is obtained at intermediate instead of low auditory SNR levels [15], which contradicts the well-known principle of inverse effectiveness. We showed that even purely temporal visual information can improve speech understanding. This was remarkable considering that this impoverished information, by itself, did not allow any identification. Only when combined with a minimum of auditory signal was identification improved and the benefits increase with increasing SNR, opposite to what one would expect from inverse effectiveness.

We then presented a simple, yet rigorous model in which auditory-visual speech perception was treated as an inference process with noisy cues. We took into account the complexity of speech by conceptualizing words as points in a multidimensional space. The behavioral data in both conditions could be fitted very well, and in particular, the largest multisensory enhancement occurred at intermediate auditory SNR. All else being equal, a decrease in the reliability of the secondary modality or an increase in the number of alternatives causes multisensory enhancement to stray further from inverse effectiveness. In spite of this breakdown, performance is completely consistent with a Bayesian model of cue integration.

Numerous studies have shown that humans are nearly Bayes-optimal in combining simple perceptual cues, even in the presence of a small conflict between the cues [16], [17], [18], [46], [47], [48], [49], [50], [51], [52], sensorimotor integration [53], [54], and other forms of cue combination [55], [56], [57], [58], [59], [60]. This suggests that in multisensory integration, Bayesian optimality is a very general principle, much more so than inverse effectiveness. Moreover, it is extremely difficult to attach any intuition to inverse effectiveness (or lack thereof), while Bayesian optimality is naturally interpreted in terms of the sharpening of probability distributions (see Figure 2).

The present model of Bayes-optimal cue combination was used to make a series of predictions. The prediction on the perception of incongruent auditory-visual stimuli was indeed confirmed by a subsequent experiment. This demonstrates the power of the model not only to explain existing results but to generalize to new situations.

### Benefits of temporal information

Previous behavioral experiments show that many forms of synchronous video can improve auditory perception: simultaneous video can reduce detection thresholds of spoken sentences in noise [61] and just seeing a speaker's head movement can improve word identification [62]. Even more strikingly, syllable identification can be improved when an identical visual stimulus is shown for different syllables [63]. The present study uses only temporal visual information and explains the enhancement effects using a probabilistic model. At a mechanistic level, we propose to attribute this set of findings to the coherent modulation of the auditory signal with facial motion. Grant and others have suggested that hearing may be improved by allowing subjects to confirm whether peaks and valleys in a noisy spectrogram belong either to foreground speech (peaks) or background noise (valleys). Coherence masking protection (CMP) and co-modulation masking release (CMR) are similar phenomena purely within the auditory modality. In the case of CMP the target signal is co-modulated across different frequency bands [64]; in the case of CMR the noise is co-modulated [65]. In either case, the co-modulation may facilitate the grouping of information as belonging to the foreground signal or background noise. For this reason the enhancement observed here with a comodulated visual stimulus may be considered a form of bimodal coherence masking protection [66].

### Comparison with other models

The model presented here has similarities to earlier probabilistic models of multisensory speech perception. In studies on the McGurk effect [43] by Massaro and colleagues (for a review, see [5]), participants had to identify a spoken syllable as, for instance, /ba/ or /da/, while both auditory and visual speech were varied on a continuum between /ba/ and /da/. The behavioral data were described well by the so-called fuzzy-logical model of speech perception (FLMP; [5], [45], [67]), in which the evidence for an alternative is expressed as a probability and the multisensory probability is obtained as the normalized product of the unisensory probabilities. The FLMP is related to Bayesian inference [45], but not equivalent to it (since it equates amounts of evidence to response frequencies, which is unjustified in a Bayesian model). Moreover, it was not known whether a Bayesian model can describe data collected with a full vocabulary.

Another predecessor is Braida's prelabeling model [32]. In this model, stimuli (consonants) are represented in a multidimensional space and “confusion matrices” reflect the uncertainty in extracting syllable identity from auditory and visual cues. Multisensory performance is computed by assuming that this space is the Cartesian product of a visual and an auditory subspace. This is different from the present model, which computes optimal multisensory performance from the product of two probability distributions in the same space. Moreover, the data available at the time were only at a few SNRs and mostly showed inverse effectiveness. The model proposed here most naturally fits with an amodal (or supramodal) word space: neither dimension of this space has a purely auditory or visual character, but instead, each sensory modality contributes some evidence in each of the feature dimensions. One possible way to think about the word space might be as the space spanned by all parameters of the production process of a word, such as the time courses of vocal chord length, lip shape, and tongue position.

The notion that words form a neighborhood relationship in some high-dimensional feature space was captured also by Luce's Neighborhood Activation Model (NAM) [28], [29]. The model uses performance measures on individual phonemes to estimate the performance of identifying full words. Similar to the present work, it incorporates word frequency (prior likelihood) and expresses lexical information as permissible points in the joint feature space. However, this model does not derive the probability of correct identification from first principles as we do here, and is based instead on a descriptive quantitative rule. Nevertheless, Auer has used this model successfully to explain performance gains in audio-visual word recognition [29]. He concludes that neighborhood relationships derived numerically from behavioral confusion matrixes can also be used to quantify audio-visual word identification performance.

### Outlook on causal inference

In the predictions above, we considered the case of integrating similar, but incongruent words, i.e. the mismatch between auditory and visual utterance is small. When more disparate word pairs are allowed, integration is no longer guaranteed. In perceptual tasks using simple stimuli, it was found that as the discrepancy increases, human subjects believe less that the two stimuli had a common source [68] and can make different responses when asked for the auditory and the visual source separately [48]. A similar effect can occur in speech perception [69], as can be experienced when watching poorly dubbed movies. Temporal discrepancy between auditory and visual speech signals also affects one's percept of unity [70]. We surmise that these results can all be modeled by a Bayesian causal inference model, in which the brain not only tries to infer stimulus identity (which word was spoken) but also whether the auditory and visual stimulus had a common source [52], [71]. The present Bayesian model could open the door to causal modeling in speech perception.

### Neural basis

The notion of inverse effectiveness was first used to describe effects seen during intracranial recordings in multisensory neurons of the superior colliculus (SC). In some of those neurons, an additional visual input was most effective at driving the cell when auditory information was poorest [14], [35], [72], [73], [74]. This pattern has also been found in multisensory neurons in the neocortex of animals [75], [76] and has been inferred in brain imaging [77], [78], although imaging data of multisensory areas have to be interpreted with great caution [79], [80]. It is important to note that inverse effectiveness on a neuronal level makes a statement about spike counts observed in a subset of multisensory neurons. Behavioral inverse effectiveness, however, is a statement about the percentage of correct behavioral responses (it has also been applied to other quantities, such as reaction times). Whether there is a connection between these measures is not clear and to our knowledge there is no rigorous work establishing such a link. In contrast, Bayes-optimal cue integration can be linked to physiology in a rigorous way, using the formalism of probabilistic population codes [81], [82], [83], [84]. The implications of this formalism for speech need to be examined in further work.

The site of multisensory integration in speech is subject of considerable debate. Common-format theories of auditory-visual speech perception suggest that modality-specific stimulus information is transformed into an amodal representation [6]. This may occur by convergence of modality-specific information onto multisensory neurons, for instance in the superior temporal gyrus/sulcus [85], [86]. This is a known convergence site for visual articulation and auditory features, and has been shown to depend on the comodulation of audiovisual stimuli [87]. Recent evidence also points at early activity (<100 ms) in the supramarginal and angular gyrus (SMG/AG) [88], [89]. Besides behavioral and fMRI data, there is ample evidence from encephalography for an early influence of the visual modality on auditory speech processing. Gamma-band activity (30 Hz or higher) associated with multimodal fusion is enhanced early after onset of congruent auditory-visual speech stimuli (30–120 ms) [90]. This effect is only observed if stimuli are simultaneous, indicating that temporal information is important for early fusion in speech. Furthermore, early auditory evoked potentials (at 50 ms) in response to speech are modulated by congruent visual stimuli [91], [92]. Taken together, the behavioral and neuro-imaging data support the notion that auditory processing itself may be aided by comodulated visual stimuli during speech perception. On the other hand, according to modality-specific theories, auditory and visual speech information is processed by modality-specific networks and then associated at a post-labeling stage [6]. Indeed, neuroimaging studies of auditory-visual speech perception implicate a variety of brain regions beyond early processing stages [93]. In general, the way auditory-visual signals are integrated remains unresolved. Further neurophysiological research is needed to constrain the possibilities on how auditory-visual integration in speech is achieved.

## Supporting Information

Figure S1 Method for generating modified video from clean audio. For details, see section 1 of the Supporting Information.
(0.56 MB TIF)

Figure S2 Variability between experiments. Auditory-visual stimuli are congruent. Visual-only performance was measured in two of these three studies. a. Identical to Figure 3a. b. Performance on the congruent trials of the second experiment (the incongruent trials were reported in Figure 7). c. Data from Ross et al., 2007
(0.11 MB TIF)

Figure S3 a. Goodness of best fit (R2) of the numerical model to the behavioral data (such as in Figure 4), for various values of vocabulary size and dimension. Negative values were set to zero for plotting purposes. In Figure 4, the parameter combination N = 2000, n = 40 was used. b. Sum squared error (on a logarithmic axis) of the analytical model as a function of dimension. The minimum is at n = 55 (fits shown in Figures 6b–c), but any sufficiently large number of dimensions allows for a good fit. A low number of dimensions does not allow for a good description of the data.
(0.20 MB TIF)

Figure S4 Optimal word recognition according to the analytical Bayesian model. a–d. Recognition performance as a function of auditory reliability, rA, for various combinations of word space dimension, n, and visual reliability, rV. Colors are as in Figure 3. Figures 6b–c were generated using the same model. Note that vocabulary size is infinite. Naturally, enhancements are larger when visual reliability is larger. e. Auditory reliability at maximum multisensory enhancement as a function of visual reliability, for fixed dimension. Lowering visual reliability causes the maximum to shift to higher values of auditory reliability. The same was shown for the numerical model in Figure 5a. f. Auditory reliability at maximum multisensory enhancement as a function of word space dimension, for fixed visual reliability.
(0.16 MB TIF)

Figure S5 Effect of an auditory word on reports of an incongruent visual word, as predicted by the Bayesian model. Experiments were simulated in which pairs of similar auditory and visual words were presented. On each trial, the observer integrates the uncertain cues and reports a single word. Frequencies of reporting the auditory word (cyan) and the visual word (magenta) are shown as a function of auditory reliability. Each plot corresponds to a given combination of vocabulary size, N, and word space dimension, n. Visual reliability was fixed at rV = 0.6. The occurrence of a maximum in the visual reports at a nonzero value of auditory reliability is consistent across vocabulary sizes and dimensions.
(0.19 MB TIF)

Supporting Information S1 (0.21 MB DOC)
