# Supplementary figures and images for: Lip-Reading Aids Word Recognition Most in Moderate Noise: A Bayesian Explanation Using High-Dimensional Feature Space
Source: PLoS One. 2009 Mar 4;4(3):e4638. doi: 10.1371/journal.pone.0004638 (PMC2645675; doi:10.1371/journal.pone.0004638)

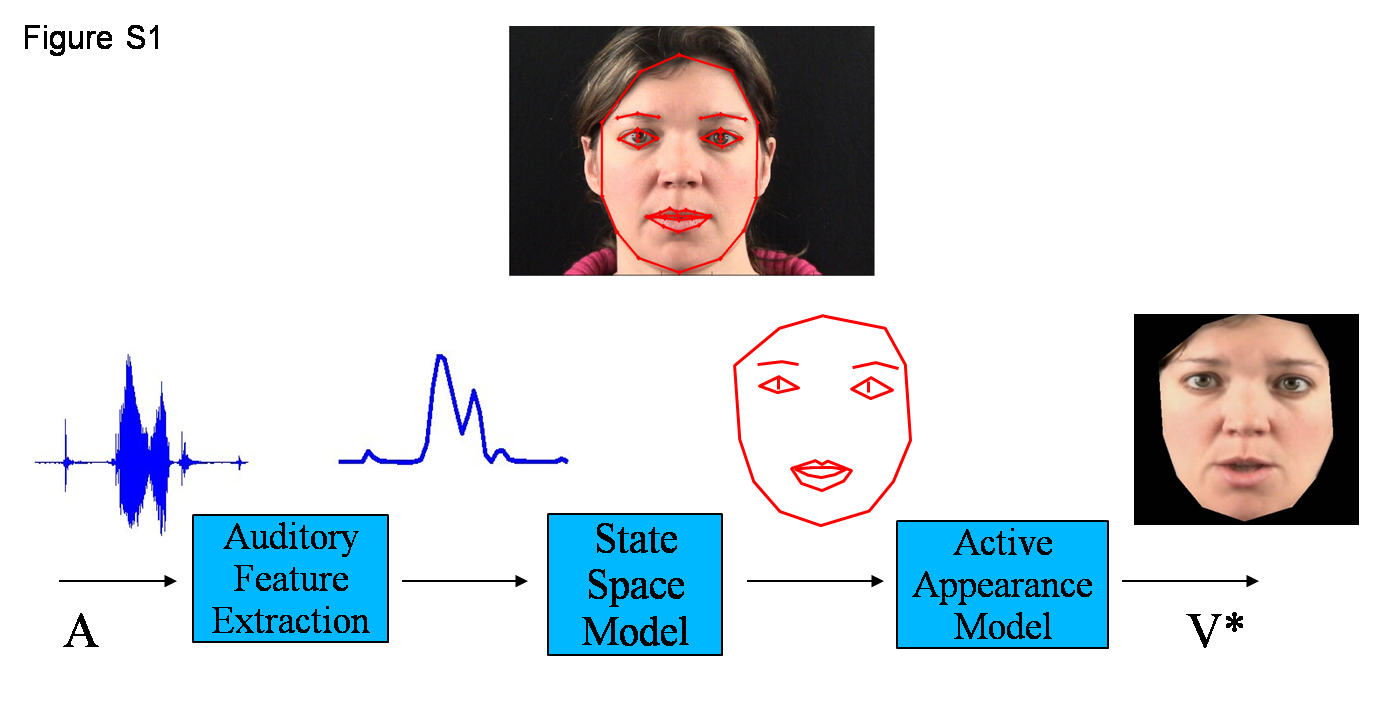

Supplement: Figure S1 — Method for generating modified video from clean audio. For details, see section 1 of the Supporting Information. (0.56 MB TIF) [file pone.0004638.s001.tif]

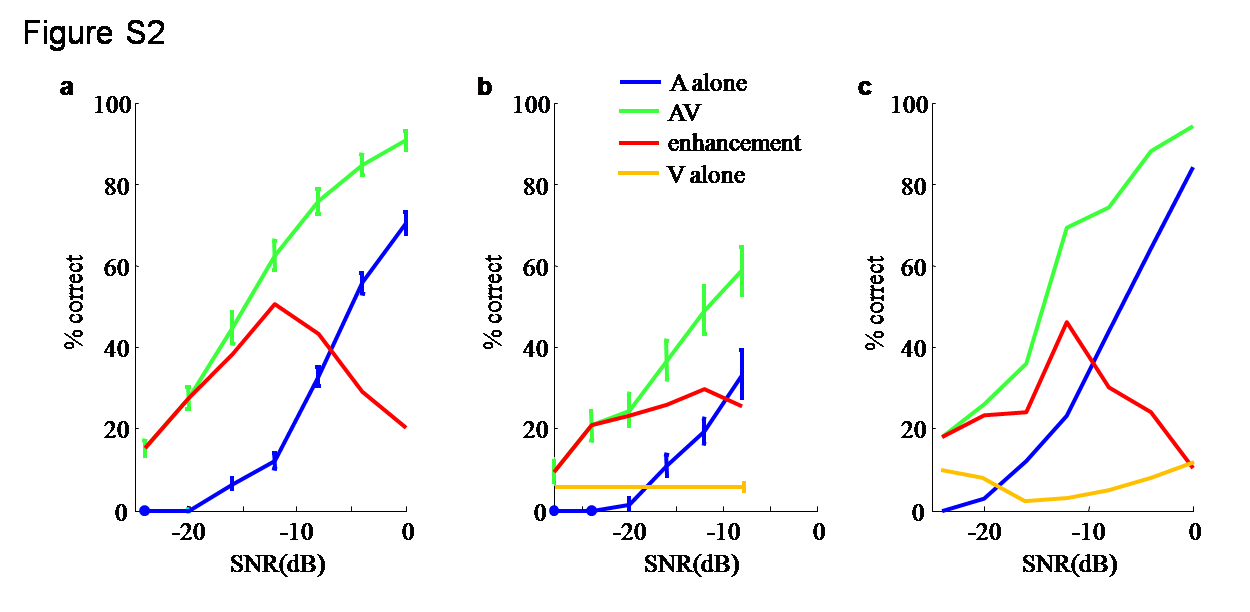

Supplement: Figure S2 — Variability between experiments. Auditory-visual stimuli are congruent. Visual-only performance was measured in two of these three studies. a. Identical to Figure 3a. b. Performance on the congruent trials of the second experiment (the incongruent trials were reported in Figure 7). c. Data from Ross et al., 2007 (0.11 MB TIF) [file pone.0004638.s002.tif]

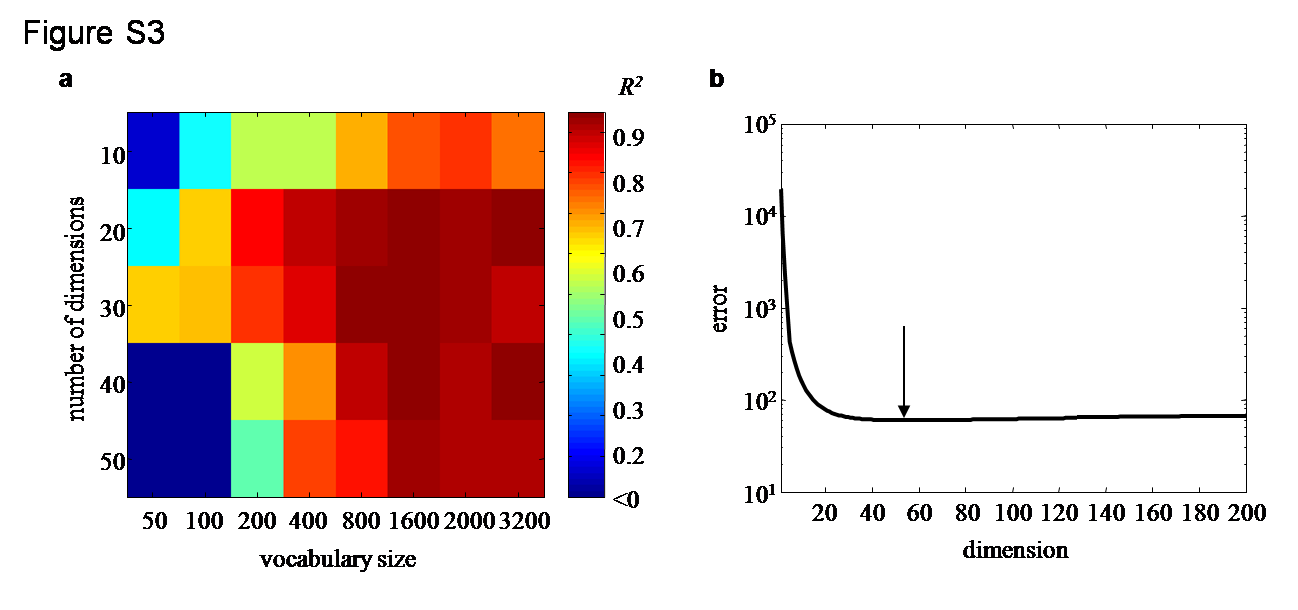

Supplement: Figure S3 — a. Goodness of best fit (R2) of the numerical model to the behavioral data (such as in Figure 4), for various values of vocabulary size and dimension. Negative values were set to zero for plotting purposes. In Figure 4, the parameter combination N = 2000, n = 40 was used. b. Sum squared error (on a logarithmic axis) of the analytical model as a function of dimension. The minimum is at n = 55 (fits shown in Figures 6b–c), but any sufficiently large number of dimensions allows for a good fit. A low number of dimensions does not allow for a good description of the data. (0.20 MB TIF) [file pone.0004638.s003.tif]

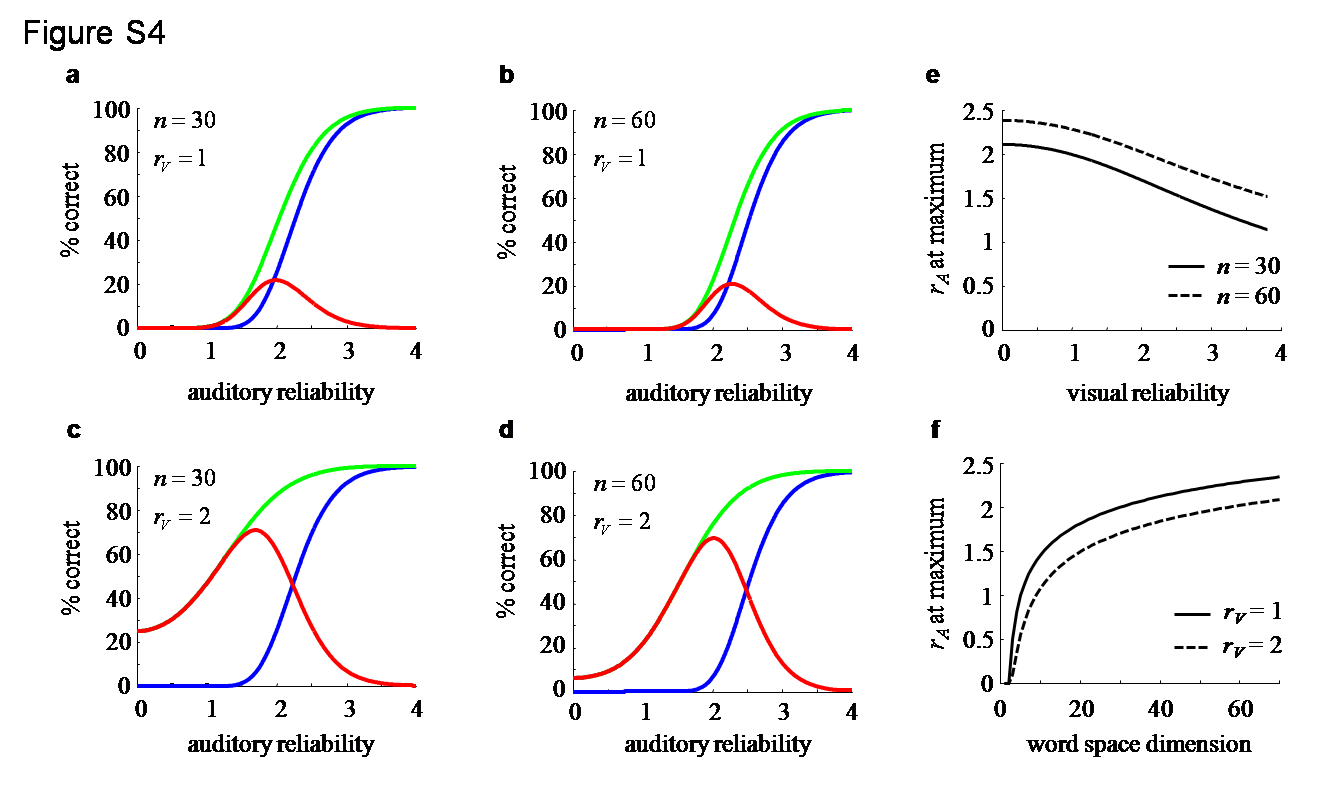

Supplement: Figure S4 — Optimal word recognition according to the analytical Bayesian model. a–d. Recognition performance as a function of auditory reliability, rA, for various combinations of word space dimension, n, and visual reliability, rV. Colors are as in Figure 3. Figures 6b–c were generated using the same model. Note that vocabulary size is infinite. Naturally, enhancements are larger when visual reliability is larger. e. Auditory reliability at maximum multisensory enhancement as a function of visual reliability, for fixed dimension. Lowering visual reliability causes the maximum to shift to higher values of auditory reliability. The same was shown for the numerical model in Figure 5a. f. Auditory reliability at maximum multisensory enhancement as a function of word space dimension, for fixed visual reliability. (0.16 MB TIF) [file pone.0004638.s004.tif]

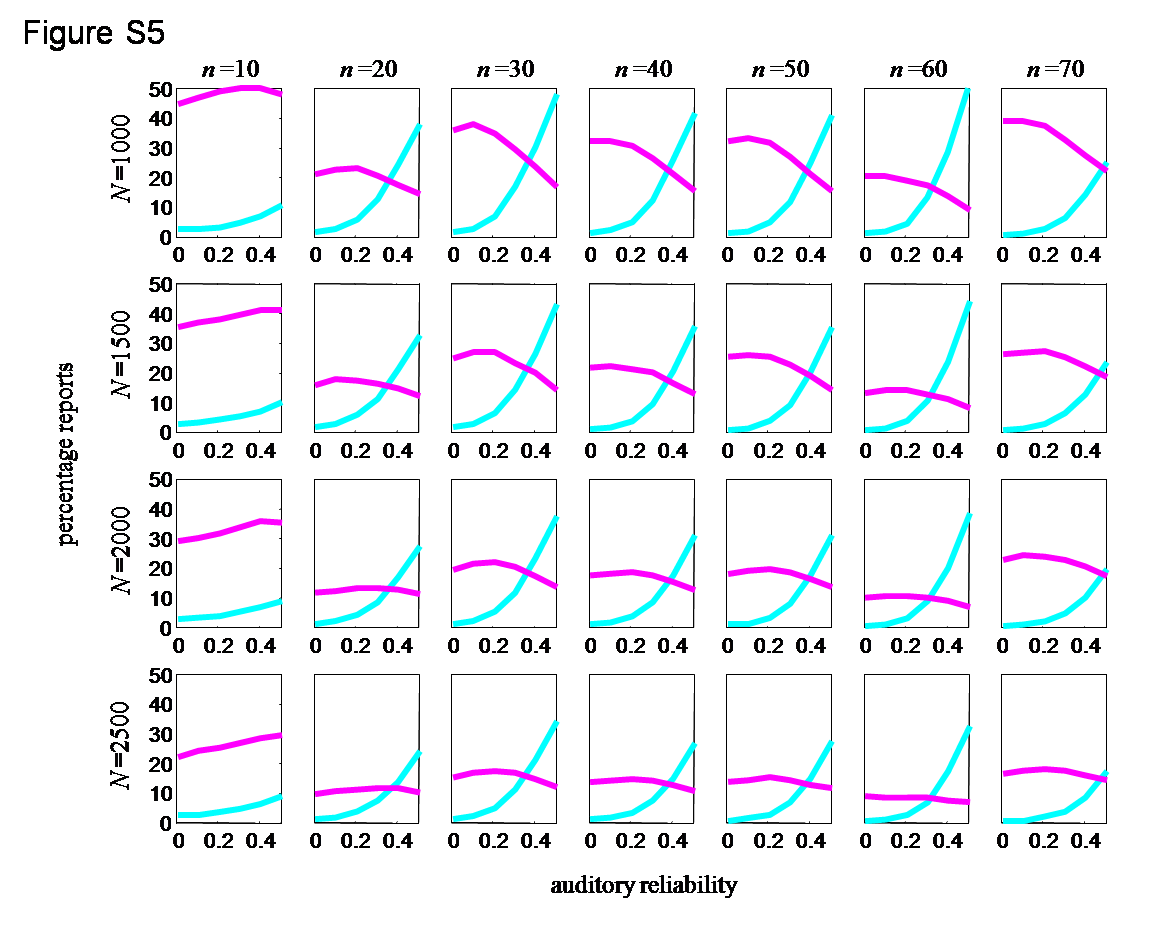

Supplement: Figure S5 — Effect of an auditory word on reports of an incongruent visual word, as predicted by the Bayesian model. Experiments were simulated in which pairs of similar auditory and visual words were presented. On each trial, the observer integrates the uncertain cues and reports a single word. Frequencies of reporting the auditory word (cyan) and the visual word (magenta) are shown as a function of auditory reliability. Each plot corresponds to a given combination of vocabulary size, N, and word space dimension, n. Visual reliability was fixed at rV = 0.6. The occurrence of a maximum in the visual reports at a nonzero value of auditory reliability is consistent across vocabulary sizes and dimensions. (0.19 MB TIF) [file pone.0004638.s005.tif]
