# Supplementary material for: Lip-Reading Aids Word Recognition Most in Moderate Noise: A Bayesian Explanation Using High-Dimensional Feature Space
Source: PLoS One. 2009 Mar 4;4(3):e4638. doi: 10.1371/journal.pone.0004638 (PMC2645675; doi:10.1371/journal.pone.0004638)
Supplement: Supporting Information S1 — (0.21 MB DOC) [file pone.0004638.s006.doc]

**Lip-reading aids word recognition most in moderate noise: a Bayesian explanation using high-dimensional feature space**

**Supporting Information**

Wei Ji Ma, Xiang Zhou, Lars A. Ross, John J. Foxe, Lucas C. Parra

This Supporting Information describes:

1. Experimental details of the AV* condition
2. Cross-study consistency
3. General theory of Bayes-optimal word recognition
4. Details of the numerical simulations
5. Details of the analytical model

# Experimental details of the AV* condition

In the AV* condition, we intended to decrease the amount of information provided by the visual stimulus while preserving as much as possible the appearance of the talking face (see Figure S1). A natural appearance of the visual stimulus has been found to be important for effective AV fusion of speech (Schwartz JL et al., 2004). To this end, we used an “Active Appearance Model” (AAM) – a computer program that can generate images of faces when provided with a set of landmark points indicating locations and shape of the lips, eyes, brows, and an outline of the face (Cootes TF et al., 2001). For a given speaker, the locations of such points covary with auditory features in a predictive fashion.

Therefore, by using auditory features such as the power of different frequency bands, one can reproduce with some accuracy the locations of these points. For this, a second computer program is used which is based on a “State Space Model” (Lehn-Schioler T, 2005). The parameters of both these models are adjusted (trained) on a set of example images. We used this technique to generate an artificial video of a talking face by using only the total instantaneous power of the audio signals. The SSM was trained to generate landmark points from the audio power envelope using manually labeled landmark points (50 in our case) for a set of 100 images (4 word utterances of 1 s duration at 25 frames/s) and the associated audio signal power measured in corresponding 40-ms time windows. The AAM was trained to generate video frames from these landmark points on the same set of images. Once trained, these two programs (SSM and AAM) were then used to generate artificial video from the power of the clean speech signal for all 500 test words.

Sample stimuli can be found on

<http://bme.engr.ccny.cuny.edu/faculty/parra/bayes-speech/>

# Cross-study consistency

Behavioral performance varies depending on the specifics of the experimental protocol. We compared the behavioral results obtained by (Ross LA et al., 2007) and the two instantiations of the A and AV conditions in the two experiments reported here (see Figure S2). In all three instances, the stimuli were selected from the same video recordings and presented in a similar fashion. The experiments differed in the way SNR was controlled and in the additional conditions presented to the subject. In contrast to the study by Ross et al., we presented a static face in the A condition to control for possible benefits of focusing attention for the duration of speech presentation. Our experiments also differed in that additional stimulus conditions were interleaved (V* in the first experiment, and A≠V in the second experiment). Finally, the second experiment modulated the speech power while keeping the noise level constant to facilitate a constant effort by the subject despite the changing level of difficulty. Otherwise, when subjects notice a high level of noise they are likely to resign themselves to not understanding the word. Such a strategy, which cannot be ruled out in the first experiment, could also lead to maximum enhancement at intermediate SNR levels. The second experiment shows that the pattern of AV enhancement cannot be attributed only to strategy. These alterations of the presentation paradigm were sufficient to significantly alter the overall performance gains. However, in all three instances, the maximum gain is attained at a SNR of approximately -12 dB. While the specifics of the performance curves are likely affected by a variety of factors, the predictions of the Bayesian model are mostly qualitative and seem robust under variations.

# General theory of Bayes-optimal word recognition

The theory of how humans optimally combine uncertain pieces of sensory information is well-known but worth going through for a multidimensional problem. The first step is to specify the generative model, also called noise model. Suppose the word presented on a given trial corresponds to an *n­*-dimensional vector $\mathbf{w}$. This word generates an auditory neural response in the brain through a noisy mechanism. This response can be characterized in word space as a noise-perturbed version of the actual word $\mathbf{w}$, which we will denote $\mathbf{\mu}_{A}$. This observed utterance does not need to be an lexically correct word. For example, the word “dog” can, through articulation or neural variability, give rise to an internal representation corresponding to “gog”. We model $\mathbf{\mu}_{A}$ as being drawn from a multivariate Gaussian distribution with mean **w** and covariance matrix $\mathbf{\Sigma}_{A}$:

$p\left(\mathbf{\mu}_{A}|\mathbf{w}\right)=\frac{1}{\sqrt{\det\left(2\pi\mathbf{\Sigma}_{A}\right)}}\exp\left(-\frac{1}{2}\left(\mathbf{\mu}_{A}-\mathbf{w}\right)^{\text{T}}\mathbf{\Sigma}_{A}^{-1}\left(\mathbf{\mu}_{A}-\mathbf{w}\right)\right)$.

We assume that $\mathbf{\Sigma}_{A}$ does not depend on which word is presented. Similarly, the neural representation elicited by the visual stimulus corresponds to an utterance $\mathbf{\mu}_{V}$. We model it as being drawn from

$p\left(\mathbf{\mu}_{V}|\mathbf{w}\right)=\frac{1}{\sqrt{\det\left(2\pi\mathbf{\Sigma}_{V}\right)}}\exp\left(-\frac{1}{2}\left(\mathbf{\mu}_{V}-\mathbf{w}\right)^{\mathbf{T}}\mathbf{\Sigma}_{A}^{-1}\left(\mathbf{\mu}_{V}-\mathbf{w}\right)\right)$.

The means of both distributions are equal to the true word **w**, which indicates that on average, “dog” will indeed look and sound like “dog”.

So far we have described the generative model. The Bayesian inference process is its inverse, asking the question: given an auditory observed utterance $\mathbf{\mu}_{A}$, visual observed utterance $\mathbf{\mu}_{V}$, or both, what is the probability that the test word was **w**? The resulting probability distribution is called the posterior distribution and it is obtained using Bayes’ rule. For an auditory utterance only, this becomes:

$$p\left(\mathbf{w}|\mathbf{\mu}_{A}\right)\propto p\left(\mathbf{\mu}_{A}|\mathbf{w}\right)p\left(\mathbf{w}\right)$$

where $p\left(\mathbf{w}\right)$ is the prior distribution over words. Since only lexical words $\mathbf{w}_{i}$ (*i* = 1,…, *N*) are allowed, this prior is discrete,

$p\left(\mathbf{w}\right)=\sum_{i} \delta\left(\mathbf{w}-\mathbf{w}_{i}\right)p\left(\mathbf{w}_{i}\right)$.

As a function of **w**, $p\left(\mathbf{\mu}_{A}|\mathbf{w}\right)$ is called the likelihood function. The essence of Bayesian inference in perception is the notion that on each trial, neural activity encodes the full posterior probability distribution over the stimulus, rather than only the single most probable stimulus (the maximum-a-posteriori estimate). How this might be implemented neurally has been described elsewhere for one-dimensional stimuli (Ma WJ et al., 2006).

When there are two cues, Bayes-optimal cue integration dictates that the probabilistic information contained in both should be used in the inference process. We assume that they are conditionally independent, which means that for a given presented word, the auditory and the visual observations are subject to independent sources of noise. Under this assumption, the joint likelihood function factorizes as

$$p\left(\mathbf{\mu}_{A},\mathbf{\mu}_{V}|\mathbf{w}\right)\propto p\left(\mathbf{\mu}_{A}|\mathbf{w}\right)p\left(\mathbf{\mu}_{V}|\mathbf{w}\right)$$

Because both likelihoods $p\left(\mathbf{\mu}_{A}|\mathbf{w}\right)$and $p\left(\mathbf{\mu}_{V}|\mathbf{w}\right)$ are multivariate Gaussians, their product will be one too, with mean

$$\mathbf{\mu}_{AV}=\left(\mathbf{\Sigma}_{A}^{-1}+\mathbf{\Sigma}_{V}^{-1}\right)^{-1}\left(\mathbf{\Sigma}_{A}^{-1}\mathbf{\mu}_{A}+\mathbf{\Sigma}_{V}^{-1}\mathbf{\mu}_{V}\right)$$

and covariance matrix

MERGEFORMAT$\mathbf{\Sigma}_{AV}=\left(\mathbf{\Sigma}_{A}^{-1}+\mathbf{\Sigma}_{V}^{-1}\right)^{-1}$.

The vector $\mathbf{\mu}_{AV}$ represents the most likely utterance given the auditory and the visual observations on this trial, but like $\mathbf{\mu}_{A}$ and $\mathbf{\mu}_{V}$, it may not correspond to a lexically correct word. (As an interesting aside for the mathematically inclined reader, $\mathbf{\mu}_{AV}$ does not necessarily lie on the line connecting $\mathbf{\mu}_{A}$ and $\mathbf{\mu}_{V}$, contrary to what one might expect.) The posterior probability of word $\mathbf{w}$ based on both observations $\mathbf{\mu}_{A}$ and $\mathbf{\mu}_{V}$ on this trial is given by

$p\left(\mathbf{w}|\mathbf{\mu}_{A},\mathbf{\mu}_{V}\right)\propto p\left(\mathbf{\mu}_{A}|\mathbf{w}\right)p\left(\mathbf{\mu}_{V}|\mathbf{w}\right)p\left(\mathbf{w}\right)$.

Based on the posterior distribution $p\left(\mathbf{w}|\mathbf{\mu}_{A},\mathbf{\mu}_{V}\right)$, the subject will report the word for which this posterior probability is maximal (the maximum-a-posteriori rule):

$$\mathbf{w}_{\text{reported}}=\mathop{\text{argmax}}\limits_{\mathbf{w}} p\left(\mathbf{w}|\mathbf{\mu}_{A},\mathbf{\mu}_{V}\right)=\mathop{\text{argmax}}\limits_{\mathbf{w}} p\left(\mathbf{w}\right)\exp\left[-\frac{1}{2}\left(\mathbf{\mu}_{AV}-\mathbf{w}\right)^{\text{T}}\mathbf{\Sigma}_{AV}^{-1}\left(\mathbf{\mu}_{AV}-\mathbf{w}\right)\right]$$

Because of the discrete prior *p*(**w**), only lexically correct words will be reported. If the reported word is the same as the presented word, which we will call $\mathbf{w}_{\text{test}}$, then the trial is counted as correct. The responses to a repeatedly presented test word $\mathbf{w}_{\text{test}}$ form themselves a discrete probability distribution, the conditional response distribution $p\left(\mathbf{w}_{\text{reported}}|\mathbf{w}_{\text{test}}\right)$, which is formally given by

$$p\left(\mathbf{w}_{\text{reported}}|\mathbf{w}_{\text{test}}\right)=\iint\delta\left(\mathbf{w}_{\text{reported}}-\mathop{\text{argmax}}\limits_{\mathbf{w}} p\left(\mathbf{w}|\mathbf{\mu}_{A},\mathbf{\mu}_{V}\right)\right)p\left(\mathbf{\mu}_{A}|\mathbf{w}_{\text{test}}\right)p\left(\mathbf{\mu}_{V}|\mathbf{w}_{\text{test}}\right)d\mathbf{\mu}_{A}d\mathbf{\mu}_{V}$$

Because $\mathbf{w}_{\text{reported}}$ only depends on $\mathbf{\mu}_{AV}$ and not on $\mathbf{\mu}_{A}$ and $\mathbf{\mu}_{V}$ separately, this can be simplified to

$$p\left(\mathbf{w}_{\text{reported}}|\mathbf{w}_{\text{test}}\right)=\int\delta\left(\mathbf{w}_{\text{reported}}-\mathop{\text{argmax}}\limits_{\mathbf{w}} p\left(\mathbf{w}|\mathbf{\mu}_{AV}\right)\right)p\left(\mathbf{\mu}_{AV}|\mathbf{w}_{\text{test}}\right)d\mathbf{\mu}_{AV}$$

Now, the random variable $\mathbf{\mu}_{AV}$ is a linear combination of the random variables $\mathbf{\mu}_{A}$ and $\mathbf{\mu}_{V}$, so we can use the rules for linear combination of normally distributed variables to find that $p\left(\mathbf{\mu}_{AV}|\mathbf{w}_{\text{test}}\right)$ is a multivariate normal distribution with mean $\mathbf{w}_{\text{test}}$ and covariance matrix $\mathbf{\Sigma}_{AV}$ as defined above. Thus, it satisfies

$P\left(\mathbf{\mu}_{AV}=\mathbf{x}|\mathbf{w}_{\text{test}}\right)\propto P\left(\mathbf{\mu}_{A}=\mathbf{x}|\mathbf{w}_{\text{test}}\right)P\left(\mathbf{\mu}_{V}=\mathbf{x}|\mathbf{w}_{\text{test}}\right)$,

where the slightly different notation is needed because this is now a relationship between the probabilities densities of three random variables ($\mathbf{\mu}_{A}$, $\mathbf{\mu}_{V}$, and $\mathbf{\mu}_{AV}$) at the same value of the argument (**x**), instead of a relationship between likelihood functions for fixed values of $\mathbf{\mu}_{A}$, $\mathbf{\mu}_{V}$, and $\mathbf{\mu}_{AV}$. The one-dimensional equivalent of Eq. in terms of means and variances is the rule that is used most commonly in modeling behavioral experiments. We use it in Figure 2b. However, it should be kept in mind that it is only an “average” relation that holds for Gaussians, but not necessarily for other distributions. The more general framework is the one described here, where a Bayesian posterior (Eq. ) is computed on every trial.

It is usually impossible to obtain an analytical expression for the conditional response distribution, Eq. , since the argmax can, in general, not be simplified. Therefore, numerical simulations are needed, which will be described in the next section.

Note that the theory remains valid when a small conflict (incongruence) is introduced between the stimuli, as long as the observer still believes that the two stimuli have the same source. If this condition is violated, then using a causal-inference model is imperative (Kording KP et al., 2007).

# Details of the numerical simulations

In the simulations, words were drawn from a Gaussian distribution in *n* dimensions, centered at the origin and with covariance matrix $\mathbf{\Sigma}_{\text{word}}=c_{1}\mathbf{I}+c_{2}\mathbf{X}_{\text{word}}^{\text{T}}\mathbf{X}_{\text{word}}$. Here, **I** is the identity matrix in *n* dimensions and **X**_word_ is a 1×*n* vector with entries drawn from a Gaussian distribution with mean 0 and standard deviation 1. The locations of the words were fixed across a simulation (for fixed *N* and *n*).

The auditory variability distribution $p\left(\mathbf{\mu}_{A}|\mathbf{w}_{\text{test}}\right)$ has mean $\mathbf{w}_{\text{test}}$ and covariance matrix $\mathbf{\Sigma}_{A}=\frac{c_{3}\mathbf{D}_{A}+c_{4}\mathbf{X}_{A}^{\text{T}}\mathbf{X}_{A}}{r_{A}^{2}}$, with **D***_A_* a diagonal *n*×*n* matrix with diagonal entries drawn from a uniform distribution on [0,1]. **X***_A_* a *k*×*n* matrix (*k*<*n*) with entries drawn from a uniform distribution on [0,1]. Similarly, the visual variability distribution $p\left(\mathbf{\mu}_{V}|\mathbf{w}_{\text{test}}\right)$ has mean $\mathbf{w}_{\text{test}}$and covariance matrix $\mathbf{\Sigma}_{V}=\frac{c_{5}\mathbf{D}_{V}+c_{6}\mathbf{X}_{V}^{\text{T}}\mathbf{X}_{V}}{r_{V}^{2}}$.

Finally, a plausible prior distribution over words had to be chosen, even though the qualitative results turned out not to depend on this choice. One could use a prior corresponding to word frequencies in (spoken) English, which approximately follow a power law ((Baayen HR, 2001; Kucera H and WN Francis, 1967; Pastizzo MJ and RF Carbone Jr., 2007); see also the databases at [http://wordplay.geneseo.edu](http://wordplay.geneseo.edu/) and <http://www.psy.uwa.edu.au/mrcdatabase/uwa_mrc.htm>). However, in our experiments, no word was used more than once, and it is likely that subjects knew this to some extent. This knowledge would conflict with a prior corresponding to word frequencies in daily language, and its effect would be to flatten out the prior, making the most frequent words less likely than expected from their frequencies in spoken English. For this reason, the prior distribution was chosen to be exponential, $p\left(\tilde{w}_{i}\right)\propto e^{-\frac{i}{c_{7}}}$, where $\left\{\tilde{w}_{i}\right\}_{i=1,\ldots,N}$ is the ordering of the vocabulary according to decreasing frequency (Zipf ranking (Baayen HR, 2001)). Note that the rate of decline of the prior distribution effectively modulates vocabulary size.

A trial would proceed as follows. For a given value of auditory SNR, we compute auditory reliability $r_{A}$ using the parameters of the rectifying nonlinearity. Using $r_{A}$, we compute the auditory covariance matrix $\mathbf{\Sigma}_{A}$ and, if the trial is multisensory, the multisensory covariance matrix $\mathbf{\Sigma}_{AV}$. We then randomly select a test word from our vocabulary and draw an observed utterance $\mathbf{\mu}_{A}$ (or $\mathbf{\mu}_{AV}$) from a Gaussian distribution with mean equal to the test word and covariance matrix $\mathbf{\Sigma}_{A}$ (or $\mathbf{\Sigma}_{AV}$). We then calculate the posterior distribution and the maximum-a-posteriori estimate as described in the previous section to generate the model’s response.

For all reported simulation results, we tried different combinations of the coefficients *c*_1_ to *c*_7_, as well as different values of *k*, normal distributions for the entries of **X***_A_* and **X***_V_***,** and different prior distributions. None changed the qualitative results reported in the main text for either the congruent or the incongruent condition. Parameter *c*_1_ has to be smaller for larger *n* to generate psychometric curves that lie in a comparable range of auditory reliability. The results shown in Figures 4, 5, S3a (congruent) and 7a and S5 (incongruent) were all created with the specific choices *c*_1_ = 10/*n*, c_2_ = 0, c_3_ = 1. c_4_ = 0. c_5_ = 1, *c*_6_ = 0, and *c*_7_ = 250.

Simulations for Figure 4 (to show that the behavioral data are fit well by the Bayesian model). Vocabulary size was fixed at *N* = 2000, and dimension at *n* = 40. The relation between auditory SNR and auditory reliability was taken to be $r_{A}=\left[\alpha\left(\text{SNR}+\beta\right)\right]_{+}$, where α and β are constants and $\left[x\right]_{+}=\max\left(x,0\right)$ is a rectifying nonlinearity. First, α and β were fitted based on the behavioral data in the A condition and found to be α = 0.0343 dB^-1^ and β = 24.3 dB. Then, these values were fixed and *r_V_* were fitted, once based on the AV condition (*r_V_* = 0.559) and once based on the AV* condition (*r_V_* = 0.214). Only the group means were fit, disregarding subject variability. Goodness of fit was $R^{2}=0.917$ (average of 0.958, 0.898, and 0.894 for the A, AV, and AV* conditions respectively). All optimizations were performed using the fminsearch routine in MATLAB and with the mean squared error as cost function. In each optimization, 1000 test words were used per data point per iteration. The figure was generated using 8000 test words per data point to produce smoother traces.

Figure 4c: The simulated data used for Figure 4a were re-analyzed by dividing the vocabulary up into two groups, according to the mean Euclidean distance of each word to other words. If this mean distance was smaller than the median mean distance (computed over the entire vocabulary), the word was labeled to be in a “dense” region, otherwise in a “sparse” region. Recognition performance in A and AV conditions was computed separately for both groups.

Simulations for Figure 5 (to examine how the multisensory enhancement depends on visual reliability and vocabulary size): All parameters were identical to those used in creating Figure 4, except that in Figure 5a, visual reliability was varied, and in Figure 5b, number of words in the vocabulary was varied.

Simulations for Figure 7a and Figure S5 (prediction for incongruent stimuli): For every auditory test word, the nearest visual word was chosen (Euclidean distance) to form an incongruent word pair. Visual reliability was fixed at *r_V_* = 0.6. All other parameters were identical to those used for Figure 4, except that α and β were not used. Responses were categorized as “reporting the auditory word”, “reporting the visual word”, and “reporting some other word”. Note that unlike the congruent case, there is no notion of a “correct response”. 10,000 trials were used for each combination of vocabulary size *N*, dimension *n*, and auditory reliability *r_A_* .

Simulations for Figure S3a (to examine how the goodness of the best fit depends on vocabulary size and number of dimensions). The simulations for Figure 4 were repeated for multiple values of the vocabulary size (50, 100, 200, 400, 800, 1600, 800, 2000, and 3200) and number of dimensions (10, 20, 30, 40, and 50). The goodness of the best fit has a broad and noisy maximum between 20-50 dimension and 800-3200 words.

# Details of the analytical model

The simulations described in the previous section show how a Bayesian model correctly predicts that auditory-visual enhancement of percentage correct exhibits a maximum as a function of auditory SNR. To gain a better understanding of the robustness of this effect, we use a “toy model” which greatly oversimplifies the structure of word space, but allows us to do some analytical calculations. These will show that even this stripped-down version of the model shows the effect and can describe the behavioral data. We first analyze the model in 1 dimension, then in multiple dimensions. Throughout this section, we assume a uniform prior distribution for simplicity.

## One dimension

In 1 dimension, the equations from section 3 simplify dramatically. If the auditory and visual noise distributions in Equations and are Gaussian with standard deviations $\sigma_{A}$ and $\sigma_{V}$, respectively, then the multisensory estimates will be normally distributed with mean equal to the actual stimulus value and standard deviation equal to $\sigma_{AV}=\frac{1}{\sqrt{\sigma_{A}^{-2}+\sigma_{V}^{-2}}}$(compare Equations and ). This is smaller than both $\sigma_{A}$ and $\sigma_{V}$ and therefore, larger precision is achieved. If we define reliability as the inverse of the standard deviation, $r_{A}=\frac{1}{\sigma_{A}}$, $r_{V}=\frac{1}{\sigma_{V}}$, and $r_{AV}=\frac{1}{\sigma_{AV}}$, then precision is the square of reliability and its multisensory improvement can be expressed as $r_{AV}^{2}=r_{A}^{2}+r_{V}^{2}$. We now consider three different cases based on the number of alternatives and their spacings.

### Infinite number of equally spaced alternatives

The goal in our hypothetical one-dimensional task is to identify a stimulus from among a large number of discrete alternatives, such as determining which car from among a row of cars honked. For now, we assume that the alternatives are equally spaced and prior probabilities are uniform. In this task, the best strategy is to select on each trial the alternative that is closest to the best estimate. In Figure 6d, the vertical, dashed lines separate the regions of stimulus space in which different alternatives are chosen. The distance between the boundaries is chosen to be 2 for convenience. If the actual stimulus is the one indicated by the red dot, then the shaded area is the probability of responding correctly. The probability correct on the auditory-alone task is now equal to $p_{C}=\text{erf}\frac{r_{A}}{\sqrt{2}}$, where $\text{erf}\left(x\right)=\frac{2}{\sqrt{\pi}}\int_{0}^{x} e^{-t^{2}}dt$ is the error function. The probability correct for the optimal combination of an auditory and a visual signal is $p_{C}=\text{erf}\sqrt{\frac{r_{A}^{2}+r_{V}^{2}}{2}}$. The multisensory enhancement (ME) is

$\text{ME}\left(r_{A}\right)=p_{C}\left(AV\right)-p_{C}\left(A\right)=\text{erf}\sqrt{\frac{r_{A}^{2}+r_{V}^{2}}{2}}-\text{erf}\frac{r_{A}}{\sqrt{2}}$.

The slope of this function is given by

$\frac{d\text{ME}}{dr_{A}}\text{=}\sqrt{\frac{2}{\pi}}\frac{r_{A}}{\sqrt{r_{A}^{2}+r_{V}^{2}}}e^{-\frac{r_{A}^{2}+r_{V}^{2}}{2}}-\sqrt{\frac{2}{\pi}}e^{-\frac{r_{A}^{2}}{2}}=\sqrt{\frac{2}{\pi}}e^{-\frac{r_{A}^{2}}{2}}\left(\left(1+\frac{r_{V}^{2}}{r_{A}^{2}}\right)^{-\frac{1}{2}}e^{-\frac{r_{V}^{2}}{2}}-1\right)$.

It is easy to see that for positive $r_{V}$ this slope is always negative, and therefore the multisensory enhancement is a monotonically decreasing function. The maximum is located at the lower boundary, which is by definition at $r_{A}=0$. This shows that under the assumptions above, optimal cue combination leads to inverse effectiveness in one dimension.

### Finite number of equally spaced alternatives

Next, we try to relax the above assumptions. Suppose the number of alternatives is not infinite, but a finite number *M* as it would be for words. We still assume equal spacing. Then the correctness region for the smallest and the largest alternative is larger than for all others. Therefore, probability correct for the auditory cue has two contributions:

$p_{C}\left(A\right)=\frac{M-2}{M}\text{erf}\frac{r_{A}}{\sqrt{2}}+\frac{2}{M}\left(\frac{1}{2}\text{+}\frac{1}{2}\text{erf}\frac{r_{A}}{\sqrt{2}}\right)=\frac{1}{M}\left(1+\left(M-1\right)\text{erf}\frac{r_{A}}{\sqrt{2}}\right)$.

Similarly,

$p_{C}\left(AV\right)=\frac{1}{M}\left(1+\left(M-1\right)\text{erf}\sqrt{\frac{r_{A}^{2}+r_{V}^{2}}{2}}\right)$.

Consequently,

$$\begin{matrix} \text{ME}\left(r_{A}\right)=\frac{1}{M}\left(1+\left(M-1\right)\text{erf}\sqrt{\frac{r_{A}^{2}+r_{V}^{2}}{2}}\right)-\frac{1}{M}\left(1+\left(M-1\right)\text{erf}\frac{r_{A}}{\sqrt{2}}\right) \\ =\frac{M-1}{M}\left(\text{erf}\sqrt{\frac{r_{A}^{2}+r_{V}^{2}}{2}}-\text{erf}\frac{r_{A}}{\sqrt{2}}\right) \end{matrix}$$

which is proportional to the multisensory enhancement found for infinitely many alternatives and therefore is also monotonically decreasing.

### Finite number of alternatives with any spacings

Now let us consider the case in which the alternatives are not equally spaced. We denote the locations of the alternatives by $\left(x_{1},x_{2},\ldots x_{M}\right)$ and their distances $\Delta_{i}=x_{i+1}-x_{i}$ for $1\leq i<M$, and $\Delta_{0}=\Delta_{M}=\infty$. Then the probability correct corresponding to standard deviation $\sigma_{A}$ of the auditory estimate distribution is

$$p_{C}\left(A\right)=\frac{1}{2M}\sum_{i=1}^{M} \left(\text{erf}\frac{\Delta_{i-1}}{2\sigma_{A}\sqrt{2}}+\text{erf}\frac{\Delta_{i}}{2\sigma_{A}\sqrt{2}}\right)=\frac{1}{M}\left(1+\sum_{i=1}^{M} \frac{\Delta_{i}r_{A}}{2\sqrt{2}}\right).$$

From this we obtain the multisensory enhancement as

$$\text{ME}\left(r_{A}\right)=\frac{1}{M}\sum_{i=1}^{M} \left(\text{erf}\frac{\Delta_{i}}{2}\sqrt{\frac{r_{A}^{2}+r_{V}^{2}}{2}}-\text{erf}\frac{\Delta_{i}r_{A}}{2\sqrt{2}}\right).$$

The part in brackets is monotonically decreasing as before, and a sum of monotonically decreasing functions is also monotonically decreasing. Thus, we again find inverse effectiveness. Applying any monotonically increasing function to $r_{A}$ does not affect this result. If non-uniform prior probabilities are associated with the words, we do not know of a way to obtain the multisensory enhancement analytically. However, simulations suggest that inverse effectiveness still holds.

In short, optimal cue combination in 1 dimension shows inverse effectiveness under very general conditions. We conclude that if inverse effectiveness is not observed, as in our speech recognition experiment, the dimensionality of the space must exceed 1.

## Multiple dimensions

The same procedure can be applied to stimuli that are characterized by multiple features, but the conclusions are different. In this simple toy model, we assume that there are *n* independent features and that the observer’s vocabulary can be represented by all points on an *n*-dimensional orthogonal grid. Using this simplification, all words can be treated equally, which allows us to obtain an analytical expression for the multisensory enhancement.

### Infinite number of alternatives on a regular grid

We first consider the case in which words are arranged on a *n­*-dimensional regular (orthogonal) grid with equal distances 2 between nearest neighbors. Whereas in the one-dimensional case the correctness region was an interval of length 2, now it is a hypercube of size 2 (see Figure 6a for the 2-dimensional case). We also assume that both noise distributions are *n*-dimensional normal distributions with the same variance in all dimensions, and no covariance. Each distribution is thus characterized by only a single standard deviation. Because we assume the features are independent, the probability correct is the *n*-th power of the probability correct in 1 dimension. Thus, auditory probability correct is

$$p_{C}\left(A\right)=\left(\text{erf}\frac{r_{A}}{\sqrt{2}}\right)^{n}$$

and auditory-visual probability correct is

$p_{C}\left(AV\right)=\left(\text{erf}\sqrt{\frac{r_{A}^{2}+r_{V}^{2}}{2}}\right)^{n}$.

The multisensory enhancement is then

$\text{ME}\left(r_{A}\right)=\left(\text{erf}\sqrt{\frac{r_{A}^{2}+r_{V}^{2}}{2}}\right)^{n}-\left(\text{erf}\frac{r_{A}}{\sqrt{2}}\right)^{n}$.

Its slope is given by

$$\begin{matrix} \frac{d\text{ME}}{dr_{A}}=n\left(\text{erf}\sqrt{\frac{r_{A}^{2}+r_{V}^{2}}{2}}\right)^{n-1}\sqrt{\frac{2}{\pi}}\frac{r_{A}}{\sqrt{r_{A}^{2}+r_{V}^{2}}}e^{-\frac{r_{A}^{2}+r_{V}^{2}}{2}}-n\left(\text{erf}\frac{r_{A}}{\sqrt{2}}\right)^{n-1}\sqrt{\frac{2}{\pi}}e^{-\frac{r_{A}^{2}}{2}} \\ =\sqrt{\frac{2}{\pi}}ne^{-\frac{r_{A}^{2}}{2}}\left(\left(\text{erf}\sqrt{\frac{r_{A}^{2}+r_{V}^{2}}{2}}\right)^{n-1}\frac{r_{A}}{\sqrt{r_{A}^{2}+r_{V}^{2}}}e^{-\frac{r_{V}^{2}}{2}}-\left(\text{erf}\frac{r_{A}}{\sqrt{2}}\right)^{n-1}\right) \end{matrix}$$

For sufficiently large *n,* this function is not monotonically decreasing. Instead, its maximum is obtained by setting the slope to 0:

$$\begin{matrix} \frac{d\text{ME}}{dr_{A}}=0\Leftrightarrow\left(\text{erf}\sqrt{\frac{r_{A}^{2}+r_{V}^{2}}{2}}\right)^{n-1}\frac{r_{A}}{\sqrt{r_{A}^{2}+r_{V}^{2}}}e^{-\frac{r_{V}^{2}}{2}}-\left(\text{erf}\frac{r_{A}}{\sqrt{2}}\right)^{n-1}=0 \\ \left(1+\frac{r_{V}^{2}}{r_{A}^{2}}\right)\left(\frac{\text{erf}\frac{r_{A}}{\sqrt{2}}}{\text{erf}\sqrt{\frac{r_{A}^{2}+r_{V}^{2}}{2}}}\right)^{2\left(n-1\right)}=e^{-r_{V}^{2}} \end{matrix}$$

This equation has a unique positive solution for $n>2$, which is plotted as a function of *n* in Figure S4f for two values of $r_{V}$. The location of the maximum is rapidly shifting towards higher values as the dimension increases. In dimensions 1 and 2, the equation has no solution and therefore inverse effectiveness is predicted.

### Finite number of alternatives on a regular grid

So far, we have assumed an infinite grid. However, the number of common monosyllabic words, although large, is finite. Therefore we consider a case in which words are arranged on the vertices of an *n*-dimensional regular grid of size $M\times M\times\cdots\times M$. The auditory probability correct is a direct generalization of the expression found in section 5.1.2:

$p_{C}\left(A\right)=\left(\frac{1}{M}\left(1+\left(M-1\right)\text{erf}\frac{r_{A}}{\sqrt{2}}\right)\right)^{n}$.

The multisensory enhancement is

$\text{ME}\left(r_{A}\right)=\left(\frac{1}{M}\left(1+\left(M-1\right)\text{erf}\sqrt{\frac{r_{A}^{2}+r_{V}^{2}}{2}}\right)\right)^{n}-\left(\frac{1}{M}\left(1+\left(M-1\right)\text{erf}\frac{r_{A}}{\sqrt{2}}\right)\right)^{n}$.

The qualitative properties of this function are very similar to those of the one for an infinite grid. In analogy to section 5.1.3, one can also relax the assumption of regularity and instead assume a general rectilinear grid. We do not go through that calculation here as it is just a minor variation of the above.

We conclude that in higher dimensions, optimal cue combination does not lead to inverse effectiveness, but to maximum effectiveness when reliability and performance in the primary modality take on intermediate values.

## Simulations

Simulations for Figure 6b-c: We fitted the analytical model to the behavioral data in the A, AV and AV* conditions. As in the numerical simulations, we assumed a threshold-linear relationship between auditory reliability and SNR: $r_{A}=\left[\alpha\left(\text{SNR}+\beta\right)\right]_{+}$. There are five free parameters in the model: *α*, *β*, the dimension *n*, and both visual reliabilities, $r_{V}$ for AV and $r_{V*}$ for AV*. (Note that vocabulary size is infinite in this model.) All of these except for *n* are expected to vary between subjects. Since data were insufficient to fit four parameters for an individual subject, we used a different approach. For each value of *n* between 1 and 1000, we first fitted *α*, *β*, $r_{V}$, and $r_{V*}$ to the group data (percentages correct averaged over all subjects) by minimizing sum squared error using “fminsearch” in Matlab. We chose the value of *n* for which the error was minimal, and fixed *α* and *β* at their corresponding best values. Then, for these values of *n*, *α*, and *β*, we fitted $r_{V}$, and $r_{V*}$ for each subject individually.

We found the dimension with the lowest error for the group data fit to be *n* = 55, but the error was more or less the same for any sufficiently large *n* (see Figure S3b). For small values of the dimension (about *n* < 30), the model cannot describe the data well. For *n* = 55, we obtained *α* = 0.0566 dB^-1^ and *β* = 48.9 dB from the group fit, and $r_{V}=1.55\pm0.05$, and $r_{V*}=0.58\pm0.08$ from the individual subjects’ fit. The latter two values are consistent with the fact that the AV* condition provides less visual information. The average fit was very good ($R^{2}=0.97; 0.99; 0.97$ for A, AV, and AV* respectively) and is shown in Figures 6b and 6c. Given the simplifications of the model and the assumptions in the fitting procedure, these fits cannot be taken as strong evidence for the particular parameter values. However, they show that it is possible to fit a very basic version of the optimal cue integration model to human speech perception data, and that the characteristics of the data are a consequence of the high dimension of speech space.

## Incongruent stimuli

Within the analytical mode, we can consider integration of cues produced by similar, but slightly incongruent stimuli, such as auditory “bay” and visual “pay”. We assume that such similar stimuli are two neighboring points on the grid. Thus, they are identical in all dimensions except for one. We now first consider this dimension. Without loss of generality, we set the location of the auditory stimulus to 0 and the location of the visual stimulus to 2. The auditory estimate distribution is Gaussian with mean 0 and inverse standard deviation $r_{A}$. The visual estimate distribution is Gaussian with mean 2 and inverse standard deviation $r_{V}$. We assume that $r_{A}$ and $r_{V}$ are not so large that the stimuli are perceived as being in conflict. Instead, they will get integrated. The auditory-visual estimate distribution is Gaussian with mean (Ernst MO and MS Banks, 2002; Yuille AL and HH Bulthoff, 1996)

$$\mu_{AV}=\frac{2r_{V}^{2}}{r_{A}^{2}+r_{V}^{2}}$$

and inverse standard deviation $\sqrt{r_{A}^{2}+r_{V}^{2}}$. There are now three possible response categories:

- The subject reports the auditory word.
- The subject reports the visual word.
- The subject reports another word.

In this dimension, the probability of reporting the auditory word is equal to the probability mass in the interval $[-1,1]$, and of reporting the visual word is the probability mass in $[1,3]$. These probabilities are

$$\begin{matrix} \Pr\left(\text{report auditory word, 1D}\right)=\frac{1}{2}\left(\text{erf}\frac{r_{A}^{2}-r_{V}^{2}}{\sqrt{2\left(r_{A}^{2}+r_{V}^{2}\right)}}\text{+erf}\frac{r_{A}^{2}+3r_{V}^{2}}{\sqrt{2\left(r_{A}^{2}+r_{V}^{2}\right)}}\right) \\ \Pr\left(\text{report visual word, 1D}\right)=\frac{1}{2}\left(\text{erf}\frac{3r_{A}^{2}+r_{V}^{2}}{\sqrt{2\left(r_{A}^{2}+r_{V}^{2}\right)}}-\text{erf}\frac{r_{A}^{2}-r_{V}^{2}}{\sqrt{2\left(r_{A}^{2}+r_{V}^{2}\right)}}\right) \end{matrix}$$

The probability of reporting another word is found by subtracting these expressions from 1. Taking into account the other dimensions, we find

$$\begin{matrix} \Pr\left(\text{report auditory word}\right)=\frac{1}{2}\left(\text{erf}\frac{r_{A}^{2}-r_{V}^{2}}{\sqrt{2\left(r_{A}^{2}+r_{V}^{2}\right)}}\text{+erf}\frac{r_{A}^{2}+3r_{V}^{2}}{\sqrt{2\left(r_{A}^{2}+r_{V}^{2}\right)}}\right)\left(\text{erf}\sqrt{\frac{r_{A}^{2}+r_{V}^{2}}{2}}\right)^{n-1} \\ \Pr\left(\text{report visual word}\right)=\frac{1}{2}\left(\text{erf}\frac{3r_{A}^{2}+r_{V}^{2}}{\sqrt{2\left(r_{A}^{2}+r_{V}^{2}\right)}}-\text{erf}\frac{r_{A}^{2}-r_{V}^{2}}{\sqrt{2\left(r_{A}^{2}+r_{V}^{2}\right)}}\right)\left(\text{erf}\sqrt{\frac{r_{A}^{2}+r_{V}^{2}}{2}}\right)^{n-1} \end{matrix}$$

As we increase $r_{A}$, the probability of reporting an auditory word will increase monotonically. In contrast, the probability of reporting a visual word can exhibit a global maximum at nonzero values of $r_{A}$, even if *n* = 1 (and even in the extreme case of $r_{V}=0$!). These results are confirmed by numerical simulations (see Figures 7 and S5).

Baayen HR (2001) Word frequency distributions. Dordrecht: Kluwer.

Cootes TF, Edwards GJ, Taylor CJ (2001) Active Appearance Model. IEEE PAMI 23: 681-685.

Ernst MO, Banks MS (2002) Humans integrate visual and haptic information in a statistically optimal fashion. Nature 415: 429-433.

Kording KP, Beierholm U, Ma WJ, Quartz S, Tenenbaum JB, Shams L (2007) Causal inference in multisensory perception. PLoS ONE 2.

Kucera H, Francis WN (1967) Computational analysis of present-day American English. Providence, RI: Brown University Press.

Lehn-Schioler T (2005) Making Faces - State-Space Models Applied to Multi-Modal Signal Processing. In: Ph.D. Thesis.

Ma WJ, Beck JM, Latham PE, Pouget A (2006) Bayesian inference with probabilistic population codes. Nat Neurosci 9: 1432-1438.

Pastizzo MJ, Carbone Jr. RF (2007) Spoken word frequency counts based on 1.6 million words in American English. Behav Research Methods 39: 1025-1028.

Ross LA, Saint-Amour D, Leavitt VN, Javitt DC, Foxe JJ (2007) Do you see what i am saying? Exploring visual enhancement of speech comprehension in noisy environments. Cereb Cortex 17: 1147-1153.

Schwartz JL, Berthommier F, Savariaux C (2004) Seeing to hear better: evidence for early audio-visual interactions in speech identification. Cognition 93: B69-78.

Yuille AL, Bulthoff HH (1996) Bayesian decision theory and psychophysics. In: Perception as Bayesian Inference (Knill DC, Richards W, eds.). New York: Cambridge: University Press.

**Supporting Figure Captions**

**Figure S1:** Method for generating modified video from clean audio. For details, see section 1 of the Supporting Information.

**Figure S2:** Variability between experiments. Auditory-visual stimuli are congruent. Visual-only performance was measured in two of these three studies.

**a.** Identical to Figure 3a.

**b.** Performance on the congruent trials of the second experiment (the incongruent trials were reported in Figure 7).

**c.** Data from Ross et al., 2007

**Figure S3**:

**a.** Goodness of best fit (*R*^2^) of the numerical model to the behavioral data (such as in Figure 4), for various values of vocabulary size and dimension. Negative values were set to zero for plotting purposes. In Figure 4, the parameter combination *N* = 2000, *n =* 40 was used.

**b.** Sum squared error (on a logarithmic axis) of the analytical model as a function of dimension. The minimum is at *n* = 55 (fits shown in Figures 6b-c), but any sufficiently large number of dimensions allows for a good fit. A low number of dimensions does not allow for a good description of the data.

**Figure S4:** Optimal word recognition according to the analytical Bayesian model. **a-d.** Recognition performance as a function of auditory reliability, *r_A_*, for various combinations of word space dimension*,* *n,* and visual reliability, *r_V_*. Colors are as in Figure 3. Figures 6b-c were generated using the same model. Note that vocabulary size is infinite. Naturally, enhancements are larger when visual reliability is larger. **e.** Auditory reliability at maximum multisensory enhancement as a function of visual reliability, for fixed dimension. Lowering visual reliability causes the maximum to shift to higher values of auditory reliability. The same was shown for the numerical model in Figure 5a. **f.** Auditory reliability at maximum multisensory enhancement as a function of word space dimension, for fixed visual reliability.

**Figure S5:** Effect of an auditory word on reports of an incongruent visual word, as predicted by the Bayesian model. Experiments were simulated in which pairs of similar auditory and visual words were presented. On each trial, the observer integrates the uncertain cues and reports a single word. Frequencies of reporting the auditory word (cyan) and the visual word (magenta) are shown as a function of auditory reliability. Each plot corresponds to a given combination of vocabulary size, *N*, and word space dimension, *n.* Visual reliability was fixed at *r_V_* = 0.6. The occurrence of a maximum in the visual reports at a nonzero value of auditory reliability is consistent across vocabulary sizes and dimensions.
